# Prednisolone and rapamycin reduce the plasma cell gene signature and may improve AAV gene therapy in cynomolgus macaques

**DOI:** 10.1038/s41434-023-00423-z

**Published:** 2023-10-13

**Authors:** Alexander Kistner, Jessica A. Chichester, Lili Wang, Roberto Calcedo, Jenny A. Greig, Leah N. Cardwell, Margaret C. Wright, Julien Couthouis, Sunjay Sethi, Brian E. McIntosh, Kathleen McKeever, Samuel Wadsworth, James M. Wilson, Emil Kakkis, Barbara A. Sullivan

**Affiliations:** 1 Ultragenyx Pharmaceutical Inc., Novato, CA USA; 2 Gene Therapy Program, Department of Medicine, Perelman School of Medicine, University of Pennsylvania, Philadelphia, PA USA; 3 Ultragenyx Gene Therapy, Ultragenyx Pharmaceutical Inc., Cambridge, MA USA; 4 Charles River Laboratories Inc., Reno, NV USA; 5 Labcorp Drug Development, Madison, WI USA; 6 Present Address: Affinia Therapeutics, Waltham, MA USA

**Keywords:** Gene therapy, Genetic vectors, Haematological diseases

## Abstract

Adeno-associated virus (AAV) vector gene therapy is a promising approach to treat rare genetic diseases; however, an ongoing challenge is how to best modulate host immunity to improve transduction efficiency and therapeutic outcomes. This report presents two studies characterizing multiple prophylactic immunosuppression regimens in male cynomolgus macaques receiving an AAVrh10 gene therapy vector expressing human coagulation factor VIII (hFVIII). In study 1, no immunosuppression was compared with prednisolone, rapamycin (or sirolimus), rapamycin and cyclosporin A in combination, and cyclosporin A and azathioprine in combination. Prednisolone alone demonstrated higher mean peripheral blood hFVIII expression; however, this was not sustained upon taper. Anti-capsid and anti-hFVIII antibody responses were robust, and vector genomes and transgene mRNA levels were similar to no immunosuppression at necropsy. Study 2 compared no immunosuppression with prednisolone alone or in combination with rapamycin or methotrexate. The prednisolone/rapamycin group demonstrated an increase in mean hFVIII expression and a mean delay in anti-capsid IgG development until after rapamycin taper. Additionally, a significant reduction in the plasma cell gene signature was observed with prednisolone/rapamycin, suggesting that rapamycin’s tolerogenic effects may include plasma cell differentiation blockade. Immunosuppression with prednisolone and rapamycin in combination could improve therapeutic outcomes in AAV vector gene therapy.

## Introduction

Adeno-associated virus (AAV) vectors are one of the most promising gene therapy tools for the treatment of rare diseases. Diseases for which AAV vector–mediated therapies are in clinical development include retinal dystrophies, hemophilia A and B, muscular dystrophies, and metabolic diseases [1–14]. The beneficial properties of AAV vectors include broad tissue tropism, ability to transduce nondividing cells, and long-term gene expression [15]. A culmination of clinical successes in AAV gene therapy is the approval in recent years of AAV-based therapeutics by the FDA and EMA [16–19].

Despite the achievements of AAV gene therapy thus far, a major obstacle that remains is activation of de novo and preexisting immunity toward the capsid and encoded transgene antigens, which alter therapeutic efficacy and can lead to toxicity [20, 21]. Preexisting neutralizing antibodies (NAbs) against AAV are commonly found in humans after exposure to wild-type AAV early in life, resulting in broad cross-reactivity across AAV serotypes of different origins [22–28]. NAbs can neutralize vectors at relatively low titers and abrogate transduction, largely constituting an efficacy issue rather than a safety issue [29–33]. T-cell responses directed to AAV capsids have also been demonstrated, hypothetically leading to destruction of transduced host cells and a decline in transgene expression [4, 31, 34–37]. Expression of the therapeutic protein can induce T-cell responses [38–40], a potential issue in patients with a nonfunctional gene or who are gene deficient, where central tolerance to the transgene is absent [41, 42]. Additionally, innate immunity plays an important role in promoting anti-capsid and anti-transgene immune responses after AAV vector administration, including type I interferons (IFNs), TLR9, TLR2, and mitochondrial antiviral-signaling protein [43–48].

A number of strategies have been explored to circumvent the different components of AAV gene therapy immunity to improve therapeutic outcomes. In nonhuman primates (NHPs), depletion of anti-AAV antibodies can be achieved by treatment with the IgG-degrading enzyme of *Streptococcus pyogenes* (IdeS) or by multiple rounds of plasmapheresis [49–51]. In addition, CpG-depleted vector genomes have been used to avoid chronic activation of innate immunity [52–55]. Glucocorticoids have been broadly applied in clinical trials both reactively and prophylactically to control anti-vector immune responses and improve transgene expression [8, 10, 11, 56]. However, in one trial, glucocorticoid treatment did not stabilize transgene expression [57], and in another trial, some patients required transition from glucocorticoids to glucocorticoid-sparing immune-modulating agents to enable maintenance of transgene expression and eventual weaning from all immune-modulating agents [58]. Targeting multiple aspects of the immune system is therefore likely to be important in managing AAV vector immunogenicity, and as such, combinations of immunosuppressive drugs targeting both T cells and B cells, such as rapamycin and rituximab, are being explored in preclinical models and in the clinic [59–65].

Extensive research over a number of years has led to important advances regarding AAV vector immunogenicity; however, it remains unclear which strategy or combination of strategies is best able to circumvent the host immune response and facilitate optimal gene therapy effectiveness. In the current report, novel combinations of prophylactic immunosuppression (IS) regimens were tested in two male cynomolgus macaque studies using an AAVrh10 vector expressing human coagulation factor VIII (hFVIII). Study 1 compared no IS with rapamycin, azathioprine, and cyclosporin A in combination; rapamycin and cyclosporin A in combination; and prednisolone. The azathioprine and cyclosporin A IS regimen previously demonstrated tolerance induction in a canine model of mucopolysaccharidosis I using enzyme replacement therapy [66]; antibody production and class switching was prevented and maintained for up to 6 months, which allowed for administration of enzyme replacement therapy in the absence of antigen-specific antibodies and without long-term immunosuppressive treatment. Study 2 further investigated the ability of prednisolone to suppress AAV vector immunity; no IS was compared with prednisolone, prednisolone and rapamycin in combination, and prednisolone and methotrexate in combination. The goal of the current report was to identify which of these IS treatment combinations leads to the greatest gene therapy effectiveness and, as a result, enhances AAV-mediated hFVIII gene therapy.

## Materials and methods

### NHPs and study designs

Male cynomolgus macaques were acquired from Envigo Global Services Inc. (Alice, TX, USA) and housed at Labcorp Drug Development (Madison, WI, USA) during the studies. All studies were performed according to a study protocol approved by the Labcorp study director and Ultragenyx sponsor representative, and all procedures in the protocols were approved by the Labcorp Institutional Animal Care and Use Committee. Animals were 30–56 months of age, were selected based on available results from pretest examinations (eg, body weights, food consumption, clinical observations, and clinical pathology), and were manually assigned to dose groups from the selected population based on predose social pairings. Researchers were not blinded to the treatment allocation. Standard health screening was carried out (negative for tuberculosis, simian immunodeficiency virus, simian retrovirus, simian T-lymphotropic virus type-1, B virus, external parasites, Seneca Valley virus, and malaria; vaccination or positive titer for hepatitis A and measles). In study 2, additional screening was conducted for gastrointestinal (GI) parasites and bacteria (salmonella, *Shigella*, *Yersinia*, and Chagas) within 14 days of scheduled shipment to the testing facility. Additionally, before IS treatment in study 2, animals were prophylactically treated with fenbendazole (50 mg/kg orally) once daily for 3 days to eliminate traces of GI parasites that may have been below the threshold of detection of the health screening assays.

All animals had initial NAb titers of ≤1:5 to AAVrh10 capsid, determined as previously described [23, 67]. Thirty-four male cynomolgus macaques were administered a single intravenous dose of 1 × 10^13^ genome copies/kg of AAVrh10.EnTTR.TTR.hFVIIIco-SQ.PA75.

**Fig. 1:**
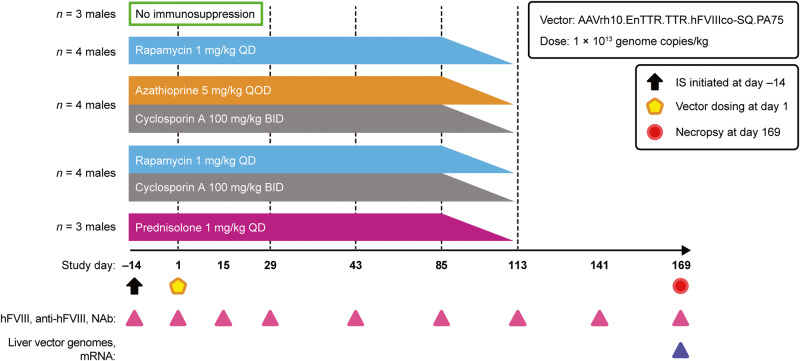
Study 1 design. Male cynomolgus macaques were administered intravenously with 1 × 10^13^ genome copies/kg of AAVrh10.EnTTR.TTR.hFVIIIco-SQ.PA75 with no IS or the following IS regimens: rapamycin alone (1 mg/kg QD), azathioprine (5 mg/kg QOD)/cyclosporin A (100 mg/kg BID), rapamycin (1 mg/kg QD)/cyclosporin A or prednisolone (1 mg/kg QD) alone. IS regimens were initiated 14 days before vector administration and tapered 25% per week between days 85 and 113. hFVIII plasma expression, anti-hFVIII IgG titers, anti-AAVrh10 NAb titers, and liver hFVIII genome copies and transcript RNA levels were evaluated in individual macaques at the indicated time points. AAV adeno-associated virus, BID twice a day, hFVIII human coagulation factor VIII, IS immunosuppression, NAb neutralizing antibody, QD once a day, QOD every other day.

In study 1, three animals received oral sterile water for irrigation (no IS group), four animals received oral rapamycin, four animals received oral azathioprine and cyclosporin A (azathioprine/cyclosporin A), four animals received oral rapamycin and cyclosporin A (rapamycin/cyclosporin A), and three animals received oral prednisolone (Fig. 1). Blood samples were taken at day –14 and then at days 1, 15, 29, 43, 85, 113, 141, and 169. Two animals from the rapamycin group were euthanized at days 68 and 75, and all four animals from the rapamycin/cyclosporin A group were euthanized at days 38, 45, 57, and 57 (Supplementary Fig. S1). All other animals from study 1 were euthanized and necropsied at day 169 per the study protocol.

**Fig. 2:**
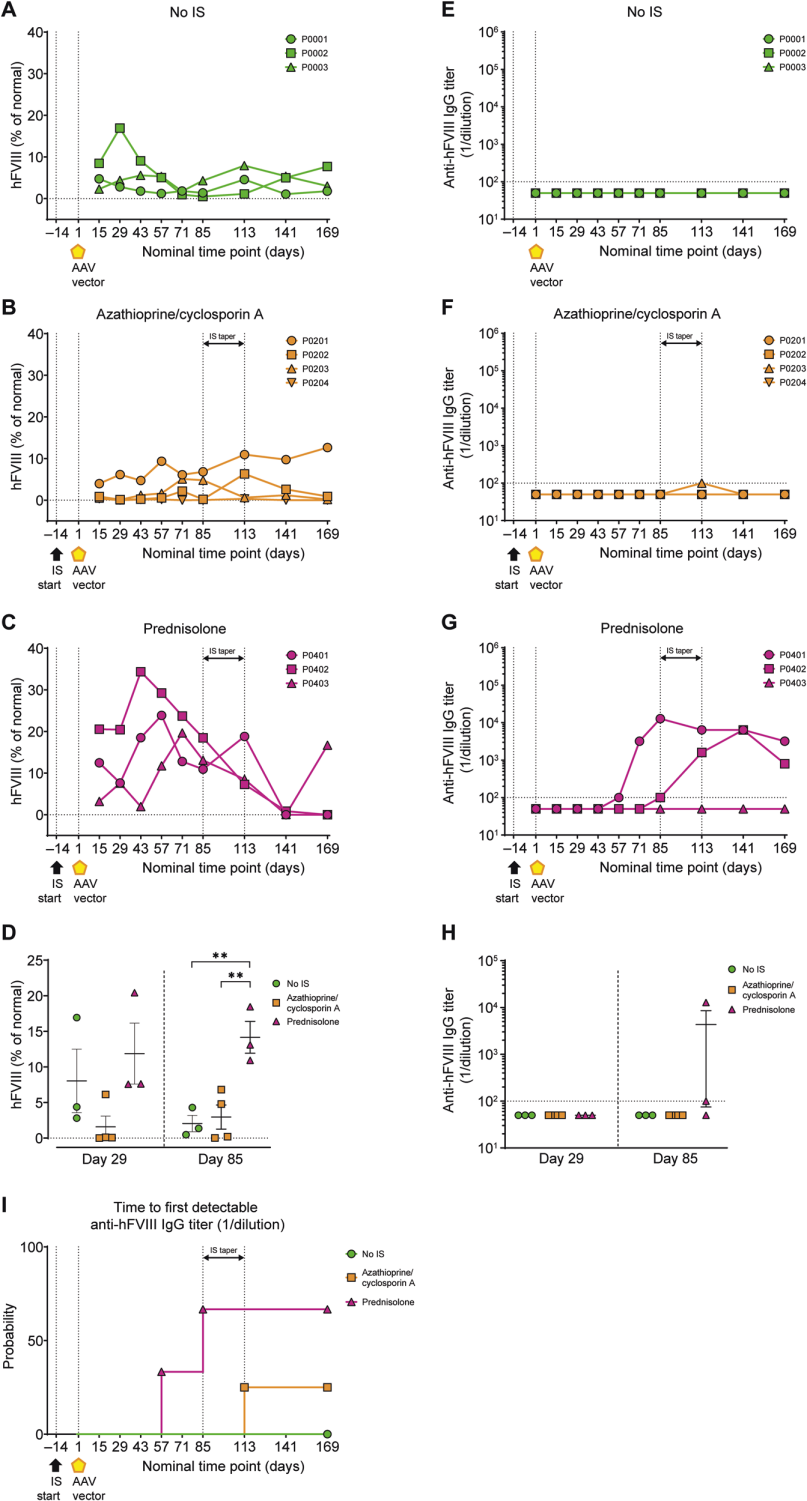
Prophylactic prednisolone increases peripheral blood transgene expression, but effects are lost upon taper. Plasma hFVIII levels and serum anti-hFVIII IgG titers were evaluated in male cynomolgus macaques by ELISA. **A**–**C** hFVIII levels and **E**–**G** anti-hFVIII IgG titers are shown for each individual cynomolgus macaque at all time points analyzed. **D** hFVIII levels and **H** anti-hFVIII IgG titers at days 29 and 85. Data are represented as mean value per group ±SEM. **I** Time to first detectable anti-hFVIII IgG titers (≥10^2^). Horizontal dashed lines in **A**–**D** at hFVIII (% of normal) = 0 indicate assay limit. Horizontal dashed lines in **E**–**H** at anti-hFVIII IgG titer (1/dilution) = 10^2^ indicate assay limit of detection; samples below limit of detection are plotted as half the assay limit. Vertical dashed lines in **A**–**C**, **E**–**G**, and **I** at days –14 and 1 indicate start of IS regimen and AAV administration, respectively. Vertical dashed lines in **A**–**C**, **E**–**G**, and **I** at days 85 and 113 indicate IS taper. Statistical analysis in **D** was performed using Kruskal–Wallis test with Dunn’s multiple comparisons test for day 29 and ordinary one-way ANOVA with Tukey’s multiple comparisons test for day 85; and in **H** was performed using Kruskal–Wallis with Dunn’s multiple comparisons test for day 85. **p < 0.01. See also Supplementary Fig. S3. AAV adeno-associated virus, hFVIII human coagulation factor VIII, *IS* immunosuppression.

In study 2, four animals received oral sterile water for injection (no IS group), four animals received oral prednisolone, four animals received oral prednisolone and oral rapamycin (prednisolone/rapamycin), and four animals received oral prednisolone and subcutaneous methotrexate (prednisolone/methotrexate) (Fig. 2). Blood samples were taken at day 1 and then at days 6, 15, 29, 43, 71, 99, 127, 155, 183, and 217. The animals were removed from the study and were repurposed for a follow-up gene therapy study and euthanized thereafter on day 364 or 427.

### IS protocols

For study 1, IS regimens consisted of oral rapamycin (RAPAMUNE; Pfizer, New York, NY, USA; 1 mg/kg daily), azathioprine (Alfa Aesar, Tewksbury, MA, USA; suspended in Ora-Blend; 5 mg/kg every other day), cyclosporin A (CycloSPORINE; Teva Pharmaceuticals USA, Inc., North Wales, PA, USA; 50 mg/kg twice a day), and prednisolone (Pharmaceutical Associates Inc., Greenville, SC, USA; 1 mg/kg daily). In the rapamycin/cyclosporin A group, rapamycin was administered 4 h after the first cyclosporin A treatment to mitigate interactions between the two drugs [68]. IS regimens began 14 days before vector administration and were tapered from day 85 at 25% per week for 28 days.

For study 2, IS regimens consisted of oral prednisolone (Pharmaceutical Associates Inc.; 1 mg/kg daily), rapamycin (RAPAMUNE; Pfizer; 1 mg/kg daily), and subcutaneous methotrexate (Fresenius Kabi USA LLC, Lake Zurich, IL, USA; 0.4 mg/kg weekly). Rapamycin was dose reduced to maintain a trough level in the blood of ~4–8 ng/mL before vector administration on day 15. Rapamycin levels were measured by liquid chromatography/tandem mass spectrometry in EDTA-treated whole blood at Labcorp Drug Development. IS regimens began 14 days before vector administration. Prednisolone was tapered between days 43 and 71 at 25% per week. Rapamycin and methotrexate were tapered between days 99 and 127 at 25% per week.

### Measurement of fibrinogen in NHP plasma

Blood was collected and processed to plasma per Labcorp Drug Development standard operating procedures. Plasma samples were analyzed on an Instrumentation Laboratory ACL^TM^ TOP 500 CTS (Werfen, Bedford, MA, USA) using the HemosIL® Fibrinogen-C XL assay using the Clauss reference method [69].

### AAV vector production

The AAVrh10.EnTTR.TTR.hFVIIIco-SQ.PA75 used in these studies is an AAVrh10 vector containing a codon-optimized version of the hFVIII-SQ expressed from a TTR enhancer and promoter, which has been previously described [70]. In hFVIII-SQ, the B domain of hFVIII is deleted and replaced by a short 14 amino acid linker, which has been previously described [71]. AAV vectors were produced by triple transfection in HEK293 cells by the Penn Vector Core at the University of Pennsylvania (Philadelphia, PA, USA), following standard procedures and as previously described [72, 73]. For the vector used in Study 1, endotoxin was <1.0 EU/ml, purity was >96%, bioburden was negative, and vector %full was 71.3. For the vector used in Study 2, endotoxin was <1.0 EU/ml, purity was >96%, and vector %full was 66.3 (bioburden was not assessed).

### Determination of hFVIII expression in NHP plasma

hFVIII expression was measured by an ELISA as previously described [62], where all reagents were from Sigma-Aldrich (St. Louis, MO, USA) unless otherwise stated. ELISA plates were coated with anti-hFVIII IgG (Green Mountain Antibodies, Inc., Burlington, VT, USA) at a 1:500 dilution in 0.1 M of carbonate buffer (pH 9.6) and incubated overnight at 4 °C. Wells were washed four times with 0.1% Tween 20 in PBS and blocked with 5% nonfat milk (Bio-Rad Laboratories, Hercules, CA, USA) in PBS for 1 h at room temperature. After removal of the blocking buffer, plasma samples diluted in 5% nonfat milk were added to the plates in duplicate and incubated for 1 h at room temperature. A standard curve was generated for each NHP run on a plate by performing two-fold serial dilutions of hFVIII recombinant protein (Xyntha; Pfizer) in 5% nonfat milk with 4% naïve plasma from the NHP being tested. Plates were then washed four times, and sheep anti-hFVIII IgG (Thermo Fisher Scientific, Waltham, MA, USA) was added at a 1:1000 dilution in nonfat milk. After incubation for 1 h at room temperature, plates were washed four times, and HRP-conjugated anti-sheep IgG was added at a 1:1000 dilution in nonfat milk. After incubation at room temperature for 90 min, plates were washed five times and 3,3′,5,5′-tetramethylbenzidine (TMB; Seracare Life Sciences, Milford, MA, USA) was added for detection. The reaction was stopped after 5 min at room temperature using 2 N sulfuric acid, and plates were read at 450 nm using a BioTek μQuant plate reader (Winooski, VT, USA). hFVIII (% of normal) expression was normalized to the value at the day of vector dosing (day 1 for study 1 and day 15 for study 2). Each sample was measured by at least two independent assays and mean value was reported.

### Vector biodistribution

Study 1 liver samples were snap frozen at the time of necropsy, and DNA was extracted from the left lateral lobe and right medial lobe of each animal using the QIAamp DNA Mini Kit (Qiagen, Valencia, CA, USA). Detection and quantification of vector genome copies in extracted DNA were performed by quantitative PCR (qPCR) in duplicate wells, as previously described [62, 74]. Briefly, genomic DNA was isolated, and vector genome copies per µg of DNA were quantified using primers and probe designed against the hFVIII transgene sequence of the vector.

### RNA isolation and RT-qPCR

DNase-treated total RNA was isolated from 100 mg of tissue from the left lateral lobe and right medial lobe of each animal from Study 1, as previously described [62]. Following reverse transcription, qPCR was then performed on cDNA in duplicate wells with primers binding to the hFVIII transgene with TaqMan Gene Expression Master Mix for detection. RNA was measured as transcript copies/100 ng.

### Detection of anti-hFVIII IgG in NHP serum

IgG antibodies against hFVIII in NHP serum were measured by ELISA, as previously described [62]. ELISA plates were coated with 1 μg/mL of BDD hFVIII-SQ (Xyntha; Wyeth Pharmaceuticals Inc., Dallas, TX, USA) in 0.1 M of carbonate buffer (pH 9.6) and incubated overnight at 4 °C. Wells were washed five times with 0.05% Tween 20 in PBS (PBST) and blocked with 5% blotting-grade blocker (Bio-Rad Laboratories) in PBS for 1 h at room temperature. After removal of the blocking buffer, serum samples starting at a 1:100 dilution in blocking buffer were added to the plates in duplicate and incubated for 1 h at room temperature. Naïve NHP baseline serum samples were used as negative controls. Plates were then washed five times with PBST, and HRP-conjugated anti-NHP IgG (Abcam, Cambridge, MA, USA) was added at a 1:2000 dilution in blocking buffer. After incubation at room temperature for 90 min, plates were washed eight times with PBST, and then TMB (Seracare Life Sciences) was added for detection. The reaction was stopped after 5 min, and the plates were read at 450 and 550 nm using a SpectraMax M2 plate reader (Molecular Devices, San Jose, CA, USA). Values five-fold over background levels of baseline samples were considered positive, and data are reported as the endpoint titer. Samples that did not meet the positive response criteria at a 1:100 dilution were considered negative and assigned a titer of <100. Samples that were still positive at the highest dilution tested in the assay were tested again at a higher starting dilution. This process repeated until an endpoint titer for each sample was obtained. Highly positive samples were tested up to three times before an endpoint titer could be determined.

### Anti-AAVrh10 capsid antibody measurement

NAb responses against AAVrh10 were measured in serum using an in vitro HEK293 cell-based assay and lacZ-expressing vector (Vector Core Laboratory, University of Pennsylvania), as previously described [67]. The NAb titer values are reported as the reciprocal of the highest serum dilution at which AAV transduction is reduced 50% compared with the negative control. The limit of detection of the assay was a 1:5 serum dilution. Variance of this assay is ±one two-fold serum dilution due to sensitivity issues inherent to the assay [67, 75, 76].

IgG and IgM antibodies against AAVrh10 capsid in NHP serum were measured by ELISA. Ninety-six-well ELISA plates were coated with 2 × 10^9^ genome copies/well in 0.1 M of carbonate buffer (pH 9.6) and incubated overnight at 4 °C. Wells were washed three times with PBST and blocked with 5% blotting-grade blocker (Bio-Rad Laboratories) in PBS for 1 h at room temperature. After removal of the blocking buffer, serum samples diluted starting at a 1:50 dilution in blocking buffer were added to the plates in duplicate and incubated for 1 h at room temperature. Plates were then washed, and HRP-conjugated anti-NHP IgG (Abcam) or anti-NHP IgM (Alpha Diagnostic International, San Antonio, TX, USA) was added at a 1:10,000 and 1:4000 dilution, respectively, in blocking buffer. After incubation at room temperature for 1 h, plates were washed four times, and then TMB (Seracare Life Sciences) was added for detection. The reaction was stopped after 2 min at room temperature, and plates were read at 450 nm using a SpectraMax M2 plate reader (Molecular Devices). Values three-fold over background levels (baseline samples) were considered positive, and data are reported as endpoint titer. Samples that did not meet the positive response criteria at a 1:50 dilution were considered negative and assigned a titer of <1:50. Samples that were still positive at the highest dilution tested in the assay were tested again at a higher starting dilution. This process repeated until an endpoint titer for each sample was obtained. Highly positive samples were tested up to two times before an endpoint titer could be determined.

### Whole-blood IFN gene signature and plasma cell (PC) gene signature

A branched DNA (bDNA) assay was created to measure IFN type I and PC gene expression signatures. A panel of 21 IFN-α–inducible genes were selected based on a report in which whole blood from patients with systemic lupus erythematosus was used to identify an IFN-α–inducible signature to be used as a pharmacodynamic and diagnostic biomarker [77]. The PC gene signature consisted of five genes (*IGJ*, *TNFRSF17*, *IGKC*, *IGKV-4*, and *IGHA*) identified as being expressed predominantly by PCs sorted from whole blood [78]. Three housekeeping genes (*PPIB*, *HPRT*, and *POL2A)* were used for normalization and NHP pooled RNA was used as a positive control for the assay.

Whole blood was collected into PAXgene RNA tubes (BD Biosciences, San Jose, CA, USA) and frozen according to manufacturer’s instructions before shipment to Charles River Laboratories (Reno, NV, USA) for analysis. Negative and positive control samples were prepared from two male cynomolgus macaques, M1 and M2, from whole blood samples stimulated in vitro with PBS or Universal IFNα Hybrid Type 1, transferred to PAXgene RNA tubes (BD Biosciences), and frozen and shipped to Charles River Laboratories. PAXgene whole-blood RNA samples were assessed using the bDNA 30-plex assay, following the analytical method specific for this assay (development work included characterization of assay volumes and reagent dilutions). PAXgene RNA samples from the two animals were run in technical triplicate. Data from all genes were normalized to the mean of three housekeeper genes (*PPIB*, *HPRT*, and *POL2A*). The gene signature is the median of expression of these 21 genes.

PAXgene blood samples from study 2 were processed to blood lysate using the QuantiGene sample processing kit (product no. QS0111; Affymetrix, Santa Clara, CA, USA), following manufacturer’s instructions with the following augmentation. PAXgene blood and lysis mixture were incubated at 64 °C for 120 min and shaken at 350 RPM. Resulting blood lysate was diluted 1:1 with lysis mixture before freezing until bDNA analysis.

The 1:1 diluted blood lysate was analyzed using the bDNA kit (product no. QP1014; Affymetrix) and custom 30-plex panel, following the manufacturer’s instructions for blood lysate. The final plate was read on a Luminex 200 (Luminex, Austin, TX, USA) machine with the following settings (sample size = 100 µL; doublet discrimination gate = 5000–25,000; timeout = 105 s; bead region = 50). Bio-Plex Manager (Bio-Rad Laboratories) software was used to export the data into an Excel (Microsoft Corporation, Redmond, WA, USA) format to be processed for final gene signature results.

The limit of detection (LOD) for each gene was determined for each plate of bDNA analysis by taking the average raw signal of six blank wells and adding three times the SD.

$${LOD}={Average}\,{of}\,6\,{blank}\,{wells}+(3* {SD}\,6\,{blank}\,{wells})$$

Any sample below the LOD was assigned a value of LOD/2 so each sample had a numerical value. Each gene’s signal was then normalized to the geometric mean of the three housekeeping genes (*PPIB*, *HPRT1*, and *POLR2A*).

$${Reported}\,{gene}\,{value}=\frac{{Raw}\,{signal}}{{GeoMean}({raw}\,{signal}\,{{{{{\rm{PPIB}}}}}},{{{{{\rm{HPRT}}}}}}1,{{{{{\rm{POLR}}}}}}2{{{{{\rm{A}}}}}})}$$

Each individual replicate of the well was required to have a minimum bead count of 30. A bead count <30 led to a repeat analysis of that sample. Universal NHP RNA control (product no. R4534565; BioChain, Newark, CA, USA) was also plated on each plate at three concentrations (1 µg, 0.5 µg, and 0.1 µg) as an additional plate control to show a change in raw signal in correlation with decreasing concentration.

### In vitro human B-cell differentiation and IgM/IgG analysis

Peripheral blood mononuclear cells (PBMCs) from three healthy human donors (two females aged 28 and 41 years and one male aged 52 years) were obtained from STEMCELL Technologies (Vancouver, BC, Canada). Cells were plated and stimulated for 6 days with 1 µg/mL CpG oligodeoxynucleotide 2006 (InvivoGen, San Diego, CA, USA) and 100 U/mL IL-2 (R&D Systems, Minneapolis, MN, USA) and treated with 2–100 ng/mL rapamycin (STEMCELL Technologies) and/or 10–1000 ng/mL prednisolone (Sigma-Aldrich), as described by Tuijnenburg et al [79]. After 6 days, human PBMCs were resuspended in FBS staining buffer (BD Biosciences) and treated with human BD Fc Block (BD Biosciences). Cells were stained with the following fluorescently conjugated antibodies (clones) per manufacturer’s instructions: CD38 (HIT2), CD27 (MT271), IgD (IA6-2), and CD19 (HIB19) from BD Biosciences, or BCMA (REA315) from Miltenyi Biotec (Bergisch Gladbach, Germany). Intracellular staining was performed using the BD Cytofix/Cytoperm™ Fixation/Permeabilization Kit (BD Biosciences) and the following fluorescently conjugated antibodies (clones) per manufacturer’s instructions: XBP1s (Q3-695) and its corresponding isotype were obtained from BD Biosciences. S6 pS240 (REA420) and its corresponding isotype were obtained from Miltenyi Biotec. To assess lymphocyte viability, 7-aminoactinomycin D (Thermo Fisher Scientific) was used alone or in combination with cell morphology (forward scatter low, side scatter high). To assess proliferation, PBMCs were stained with 0.5 µM CFSE (Thermo Fisher Scientific) according to the manufacturer’s instructions. Analysis was performed using a Attune NXT flow cytometer (Thermo Fisher Scientific) and data analyzed using FlowJo software version 10.7.1 (Ashland, OR, USA) and GraphPad Prism version 9.0.1 (San Diego, CA, USA). Human IgM and IgG concentration in supernatants was detected with human IgG and human IgM ELISA kits from Bethyl Laboratories, Inc. (Montgomery, TX, USA) according to manufacturer’s instructions.

### In situ hybridization and quantitation

hFVIII was detected and quantified by in situ hybridization (ISH) on formalin-fixed paraffin-embedded liver samples from Study 1. Liver samples for ISH were collected from the edge and hilus regions of the right medial lobe from each animal. The liver samples were preserved in 10% neutral-buffered formalin for approximately 24 h before being transferred to 70% ethyl alcohol for 3 days prior to paraffin embedding. ISH was performed using the ViewRNA™ ISH Cell Assay system (ThermoFisher) according to the manufacturer’s protocol. Probes were made by ThermoFisher targeting unintegrated vector DNA and the codon-optimized hFVIII sequence of the transgene, and were designed so they would not cross-hybridize to any endogenous sequences in monkey tissue. Liver samples from each animal were processed for ISH, counterstained with 4′,6-diamidino-2-phenylindole (DAPI) to show nuclei and analyzed with a fluorescence microscope for the presence of Fast Red signal indicating bound probes. From each liver sample a representative image with a 20x objective was taken. Images were analyzed by HALO software (Indica Labs, v.3.6.4134.166) using the FISH-IF module (v2.2.5). The average number of hFVIII puncta per DAPI+ cell was quantified per image. Scoring criteria were also created to classify DAPI+ cells as containing zero (Score 0), 1–9 (Score 1), 10–49 (Score 2), 50–99 (Score 3), or ≥100 (Score 4) puncta of hFVIII and the average percent of DAPI+ cells falling into each of those scores was quantified.

### Statistical analysis

GraphPad Prism v.9.1.0 was used for statistical analyses. All values are expressed as mean ± SEM. The reported gene values described for the whole-blood IFN gene signature and PC gene signature were imported into MORPHEUS to generate heatmaps (https://software.broadinstitute.org/morpheus; Broad Institute, Cambridge, MA, USA). Hierarchical clustering was performed on columns, forcing groups by time, using the one minus Pearson correlation metric and the average linkage method.

## Results

### Study 1: AAVrh10.hFVIII gene therapy with prophylactic IS: rapamycin alone, prednisolone alone, or cyclosporin A in combination with azathioprine or rapamycin

#### Study design and general health outcomes

To test combination prophylactic IS regimens in AAV gene therapy, an initial study (study 1) was conducted, in which a single dose of AAVrh10.EnTTR.TTR.hFVIIIco-SQ.PA75 was administered intravenously to male cynomolgus macaques at 1 × 10^13^ genome copies/kg (Fig. 1). At screening, which was 47 days before vector administration at day 1, anti-AAVrh10 NAb titers for all animals were ≤1:5 per study inclusion criteria. Animals received the vector with no IS (*n* = 3) or one of the following four IS regimens: rapamycin (*n* = 4), azathioprine/cyclosporin A (*n* = 4), rapamycin/cyclosporin A (*n* = 4), or prednisolone (*n* = 3). To decrease potential drug–drug interactions in the rapamycin/cyclosporin A–treated animals, rapamycin was administered 4 h after cyclosporin A dosing, as previously described [68]. The IS regimens were initiated 14 days before (day –14) vector administration at day 1 and were continued until day 85 before being tapered.

Health screening and body weight analysis were carried out to assess tolerability of the IS regimens. The no IS, azathioprine/cyclosporin A, and prednisolone groups exhibited steady weight gain throughout the study (Supplementary Fig. S1). The rapamycin/cyclosporin A treatment group experienced recurrent GI-related issues and weight loss starting around day –4, before vector administration on day 1, which resulted in euthanasia for all animals in that group by day 56. The rapamycin group experienced similar GI-related issues and weight loss starting around day –4, which eventually resulted in the euthanasia of two out of four animals on day 73. *Giardia lamblia* was present in fecal cultures; therefore, the GI observations were likely due to opportunistic infections from GI parasites as a result of heavy immune suppression (data not shown). In the rapamycin/cyclosporin A–treated and rapamycin-treated animals that required early euthanasia, an increase in plasma fibrinogen was observed and was above the reference range maximum at certain time points (Supplementary Fig. S2). Due to the health issues observed in the rapamycin/cyclosporin A and rapamycin groups, data from these animals are not shown in relevant figures.

#### Levels of plasma hFVIII, transduction efficiency, and evaluation of anti-hFVIII antibodies

Plasma transgene expression, measured by percentage of normal human hFVIII levels, was detected in all study groups (Fig. 2A–D). The prednisolone group demonstrated the highest level of hFVIII in the blood during the treatment window, and at day 85, mean hFVIII expression was significantly increased in comparison with the no IS and azathioprine/cyclosporin A groups (Fig. 2D); however, hFVIII levels decreased after prednisolone taper. Plasma anti-hFVIII IgG titers were not detected in the no IS group (Fig. 2E, H, I), an unexpected result given that a previous study demonstrated 100% incidence of anti-FVIII antibodies in cynomolgus macaques after administration of an AAVrh10 vector [62]. In the prednisolone group, anti-hFVIII IgG titers were detected in two out of three animals (Fig. 2G–I), and a reduction in detectable hFVIII protein levels in plasma correlated with anti-hFVIII antibody development (Supplementary Fig. S3). One animal in the azathioprine/cyclosporin A group showed detectable anti-hFVIII IgG at day 113 only (Fig. 2F, I). Vector DNA (Supplementary Fig. S4A) and hFVIII transcript RNA levels (Supplementary Fig. S4B) were quantified in the liver at necropsy and were found to be comparable between treatment groups.

#### Analysis of anti-AAVrh10 antibodies

After vector administration, anti-AAVrh10 NAbs were detected in 100% of animals across all groups (Supplementary Fig. S5). Azathioprine/cyclosporin A and prednisolone treatment showed a trend for reduced NAbs at day 85 compared with no IS; however, this was not significant (Supplementary Fig. S5D). In two out of four azathioprine/cyclosporin A–treated animals, an increase in anti-AAVrh10 NAb titers was observed between screening and vector administration (day 1), despite these animals having exhibited NAb titers of ≤1:5 at screening (Supplementary Fig. S5B). These two animals showed reduced hFVIII expression, liver vector DNA levels, and hFVIII transcripts when compared with the azathioprine/cyclosporin A animal whose NAb titers remained ≤1:5 before vector administration. Because this readout was a cell-based assay, we investigated the possibility that the immunosuppressive drugs could cause in vitro toxicity, as previously described [80, 81]. Results of this analysis determined that, although some toxicity was observed in the assay at high concentrations of the immunosuppressive drugs, the level of immunosuppressive drugs in the NHP serum was not high enough to interfere in the in vitro AAV NAb assay (data not shown). When AAVrh10 IgM and IgG antibodies were analyzed, the azathioprine/cyclosporin A group showed reduced switching from IgM to IgG compared with the no IS control and prednisolone groups (Supplementary Fig. S6).

#### Analysis of transgene expression by ISH

For analysis of transgene expression, ISH was performed using a probe specific for hFVIII that recognizes either vector genome or hFVIII mRNA. Representative microscopic images from individual animals are shown in Supplementary Fig. S7A; quantitative image analysis results are shown in Supplementary Figs. S7B–F and Supplementary Table S1. By quantitative image analysis, the mean levels of hFVIII hybridization signal in animals in the prednisolone group were higher than in the no IS and azathioprine/cyclosporin A groups, although this did not reach statistical significance (Supplementary Fig. S7B). The proportion of DAPI+ cells containing at least one hFVIII puncta (mean ± SEM) was 61 ± 1.6%, 60 ± 13.6%, and 81 ± 1.3% in the no IS, azathioprine/cyclosporin A, and prednisolone groups, respectively; individual animal data are shown in Supplementary Table S1. Animals in the prednisolone group also demonstrated a higher proportion of cells with dense hFVIII hybridization signal (as shown by a score of 3 or 4 (50–99 and ≥100 puncta/cell, respectively) compared with animals in the no IS and azathioprine/cyclosporin A groups (Supplementary Fig. S7C). A positive visual relationship between the level of hFVIII hybridization signal and vector DNA and with hFVIII mRNA was observed, with animals with higher hFVIII hybridization signal also having higher vector DNA and hFVIII mRNA, although neither of these relationships reached statistical significance (Supplementary Figs. S7D, E).

For most animals, there was a positive visual relationship between the level of hFVIII hybridization signal on day 169 and %hFVIII in blood. The exceptions were animals P0401 and P0402. While P0401 and P0402 had high DNA, RNA and hFVIII hybridization signal in liver, hFVIII protein was undetectable in the peripheral blood. P0401 and P0402 also exhibited high levels of anti-hFVIII antibodies, suggesting that anti-hFVIII antibodies may block the ability to measure hFVIII in blood.

### Study 2: AAVrh10.hFVIII gene therapy with prophylactic prednisolone alone or in combination with rapamycin or methotrexate

#### Study design and general health outcomes

**Fig. 3:**
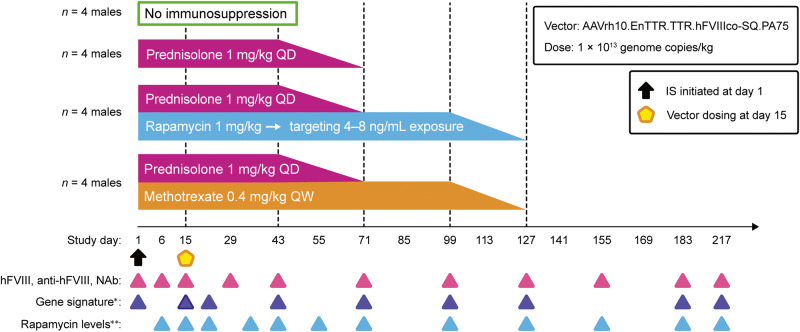
Study 2 design. Male cynomolgus macaques were administered intravenously with 1 × 10^13^ genome copies/kg of AAVrh10.EnTTR.TTR.hFVIIIco-SQ.PA75 with no IS or the following IS regimens: prednisolone alone (1 mg/kg QD), prednisolone (1 mg/kg QD)/rapamycin (1 mg/kg QD; targeting 4–8 ng/mL exposure), or prednisolone (1 mg/kg QD)/methotrexate (0.4 mg/kg QW). IS regimens were initiated 14 days before vector administration and tapered 25% per week for 4 weeks; prednisolone taper began 4 weeks after vector dosing (tapered between days 43 and 71) and the additional drugs began their taper 12 weeks after vector dosing (tapered between days 99 and 127). hFVIII plasma expression, anti-hFVIII IgG titers, anti-AAVrh10 NAb titers, gene signatures, and rapamycin levels were evaluated in individual macaques at the indicated time points. *The day 15 gene signature had three collections: one pre vector dosing, one 6 h after vector dosing, and one 24 h after vector dosing. **Rapamycin levels were measured in whole blood; levels were dose reduced to maintain a trough level of ~4–8 ng/mL before vector administration. AAV adeno-associated virus, hFVIII human coagulation factor VIII, IS immunosuppression, NAb neutralizing antibody, QD once a day, QW once a week.

Because study 1 suggested prednisolone as a promising IS regimen for AAV vector gene therapy, a second study (study 2) further investigated the efficacy of prednisolone alone or in combination with rapamycin or methotrexate using a double-taper design, where prednisolone was tapered after 1 month (day 43) and rapamycin or methotrexate were tapered after 3 months (day 99) (Fig. 3). Before study initiation, male cynomolgus macaques were screened for parasites and treated prophylactically with fenbendazole to eliminate GI parasites. Animals received 1 × 10^13^ genome copies/kg AAVrh10.EnTTR.TTR.hFVIIIco-SQ.PA75 with no IS (*n* = 4) or one of the following three IS regimens: prednisolone (*n* = 4), prednisolone/rapamycin (*n* = 4), or prednisolone/methotrexate (*n* = 4).

**Fig. 4:**
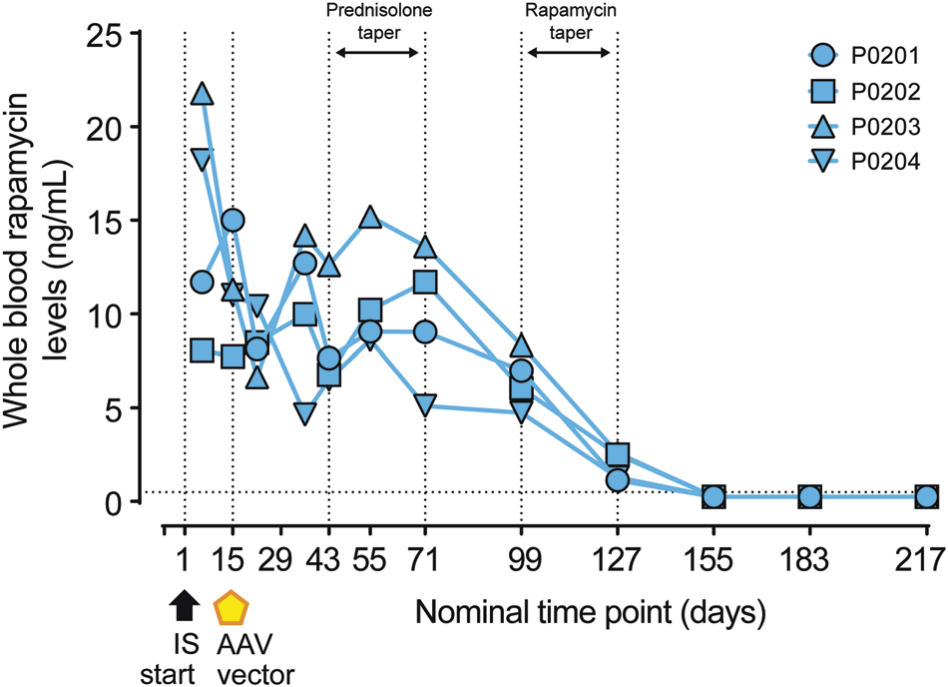
Rapamycin exposure in prednisolone/rapamycin combination group. Blood rapamycin levels were evaluated in individual male cynomolgus macaques of the prednisolone/rapamycin group by liquid chromatography/tandem mass spectrometry at the indicated time points. Horizontal dashed line at whole-blood rapamycin levels (ng/mL) = 0.5 ng/mL indicates the assay LLOQ; below quantification limit samples are plotted as half LLOQ. Vertical dashed lines at days 1 and 15 indicate start of IS regimen and AAV administration, respectively. Vertical dashed lines at days 43, 71, 99, and 127 indicate tapering of IS regimen. AAV adeno-associated virus, IS immunosuppression, LLOQ lower limit of quantification.

All IS treatments in study 2 were well tolerated, as evidenced by weight gain across all groups throughout the study duration. Overall, the prednisolone/rapamycin group gained less weight than the prednisolone/methotrexate and no IS groups (Supplementary Fig. S8). In study 2, therapeutic monitoring of rapamycin levels was carried out in whole blood of the prednisolone/rapamycin group (Fig. 4). Animals were started at 1 mg/kg rapamycin and dose reduced to achieve ~4–8 ng/mL exposure before receiving the vector on day 15. Levels ranged from 4.64 to 15.2 ng/mL between the day of vector dosing (day 15) to the beginning of rapamycin taper (day 99). Plasma fibrinogen was increased, and at certain time points was above the reference range maximum, in 3 of 4 rapamycin-treated animals and returned to baseline levels following the prednisolone and rapamycin tapers (Supplementary Fig. S9). Plasma fibrinogen was increased in one animal in the prednisolone group on day 183 due to cage-mate behavioral incompatibility.

#### Plasma hFVIII expression and evaluation of anti-hFVIII antibodies

**Fig. 5:**
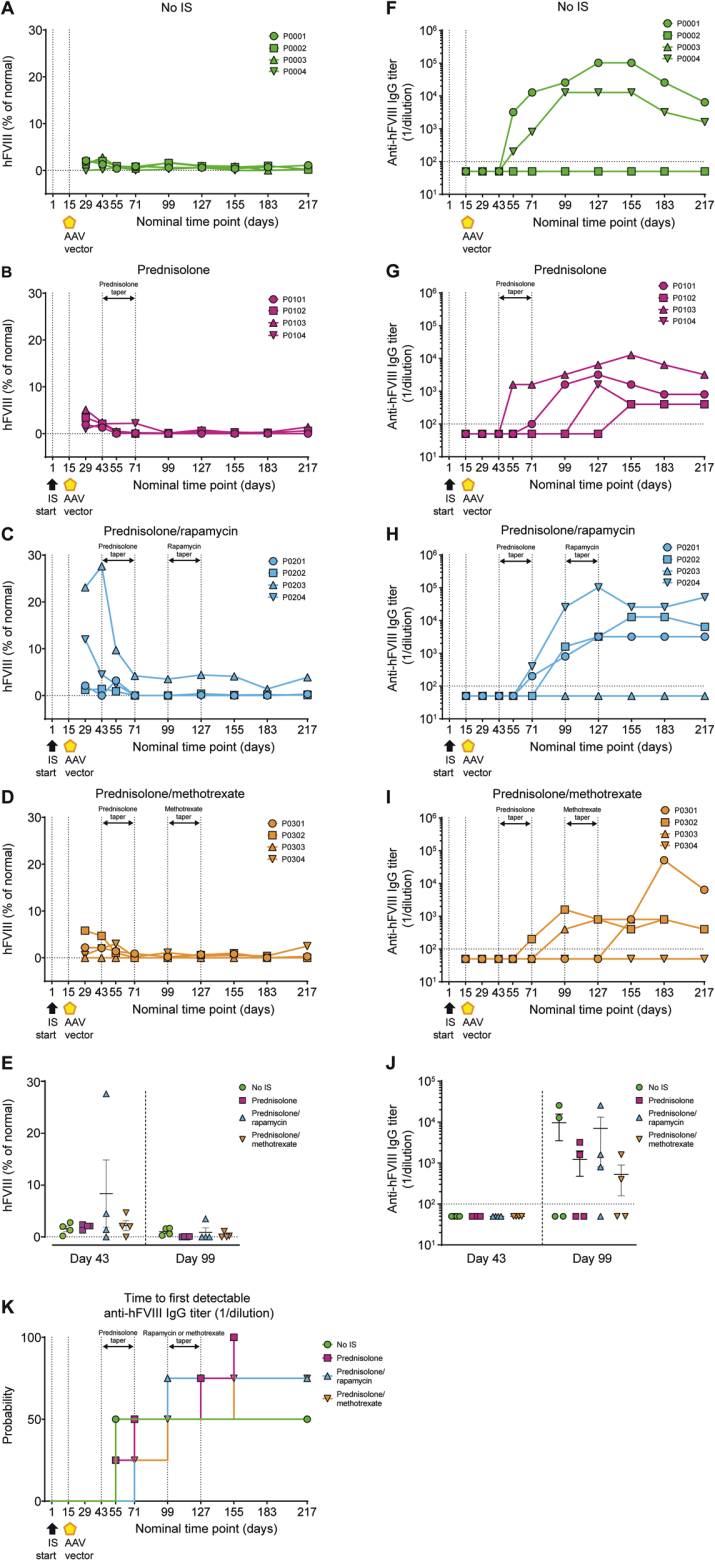
Prednisolone/rapamycin combination increases peripheral blood hFVIII transgene expression. Plasma hFVIII levels and serum anti-hFVIII IgG titers were evaluated in male cynomolgus macaques by ELISA. **A**–**D** hFVIII levels and **F**–**I** anti-hFVIII IgG titers are shown for each individual cynomolgus macaque at all time points analyzed. **E** hFVIII levels and **J** anti-hFVIII IgG titers at days 43 and 99. Data are represented as mean value per group ± SEM. **K** Time to first detectable anti-hFVIII IgG titers (≥10^2^). Horizontal dashed lines in **A**–**E** at hFVIII (% of normal) = 0 indicate assay limit. Horizontal dashed lines in **F**–**J** at anti-hFVIII IgG titer (1/dilution) = 10^2^ indicate assay limit of detection; samples below limit of detection are plotted as half the assay limit. Vertical dashed lines in **A**–**D**, **F**–**I**, and **K** at days 1 and 15 indicate start of IS regimen and AAV administration, respectively. Vertical dashed lines in **A**–**D**, **F**–**I**, and **K** at days 43, 71, 99, and 127 indicate tapering of IS regimens. Statistical analysis in **E** was performed using ordinary one-way ANOVA with Tukey’s multiple comparisons test for day 43 and Kruskal–Wallis with Dunn’s multiple comparisons test for day 99; and in **J** was performed using Kruskal–Wallis with Dunn’s multiple comparisons test for day 99; all values calculated were *p* ≥ 0.05. AAV adeno-associated virus, hFVIII human coagulation factor VIII, IS immunosuppression, LLOQ lower limit of quantification.

Analysis of hFVIII levels in the blood showed an increase in expression across all groups, with the prednisolone/rapamycin group demonstrating increased expression in two (animals P0203 and P0204) out of four animals compared with no IS, prednisolone alone, and prednisolone/methotrexate in combination (Fig. 5A–E). Moreover, one animal in the prednisolone/rapamycin group exhibited sustained hFVIII throughout the study (animal P0203) (Fig. 5C, E). Evaluation of anti-hFVIII IgG titers showed that two out of four animals from the no IS group developed anti-hFVIII antibodies from day 55 (Fig. 5F–J). Development of anti-hFVIII IgG antibodies in IS-treated animals was evident after prednisolone taper and at later time points when compared with the no IS group (Fig. 5K). In the prednisolone/methotrexate group, there was a trend for a reduced anti-hFVIII antibody response at day 99 compared with all other groups, although this was not significant (Fig. 5J).

#### Analysis of anti-AAVrh10 antibodies

**Fig. 6:**
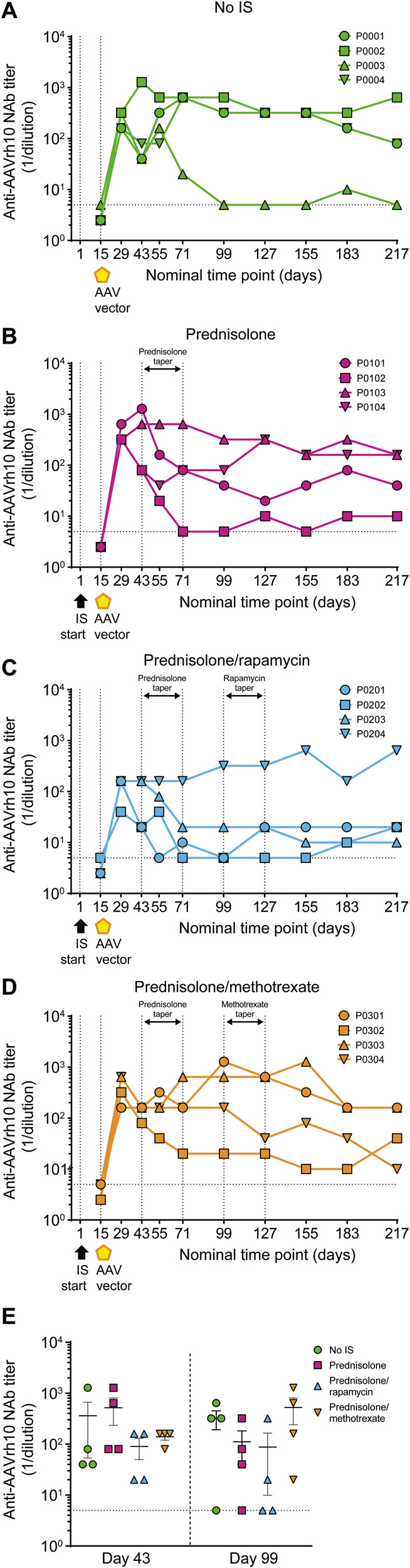
Prednisolone/rapamycin combination leads to reduction in mean anti-AAVrh10 NAb titers. Serum anti-AAVrh10 NAb titers were evaluated in male cynomolgus macaques using a cell-based neutralization assay. **A**–**D** Anti-AAVrh10 NAb titers are shown for each individual cynomolgus macaque at all time points analyzed. **E** Anti-AAVrh10 NAb titers at days 43 and 99. Data are represented as mean titer per group ± SEM. The NAb titer values are reported as the reciprocal of the highest serum dilution at which AAV transduction is reduced 50% compared with the negative control. Horizontal dashed line at anti-AAVrh10 NAb titer (1/dilution) = 5 indicates assay limit of detection; samples below limit of detection are plotted as half the assay limit. Vertical dashed lines in **A**–**D** at days 1 and 15 indicate start of IS regimen and AAV administration, respectively. Vertical dashed lines in **A**–**D** at days 43, 71, 99, and 127 indicate tapering of IS regimens. Statistical analysis in **E**, days 43 and 99, was performed using Kruskal–Wallis test with Dunn’s multiple comparisons test; all values calculated were *p* ≥ 0.05. AAV adeno-associated virus, IS immunosuppression, NAb neutralizing antibody.

After vector administration, anti-capsid AAVrh10 NAbs were detected in 100% of animals across all four groups (Fig. 6A–E). In comparison with the no IS, prednisolone alone, and prednisolone/methotrexate in combination groups, the prednisolone/rapamycin group showed a trend for reduced anti-capsid AAVrh10 NAb titers at days 43 and 99, although the difference was not significant. Notably, and in contrast to the other three groups, three out of four animals in the prednisolone/rapamycin group showed reduced levels of anti-AAVrh10 NAbs throughout the study, even after both immunosuppressive drugs were tapered (Fig. 6C, E). To evaluate whether this observed decrease in AAVrh10 NAb response also impacted responses against additional AAV serotypes, NAb titers were evaluated against AAV8 and AAV9 (Supplementary Fig. S10). Overall, the magnitude of anti-AAV8 NAb titers in the prednisolone/rapamycin group was reduced compared with the no IS and prednisolone groups, and animals treated with either prednisolone or prednisolone/rapamycin showed reduced AAV9 NAb responses when compared with no IS.

**Fig. 7:**
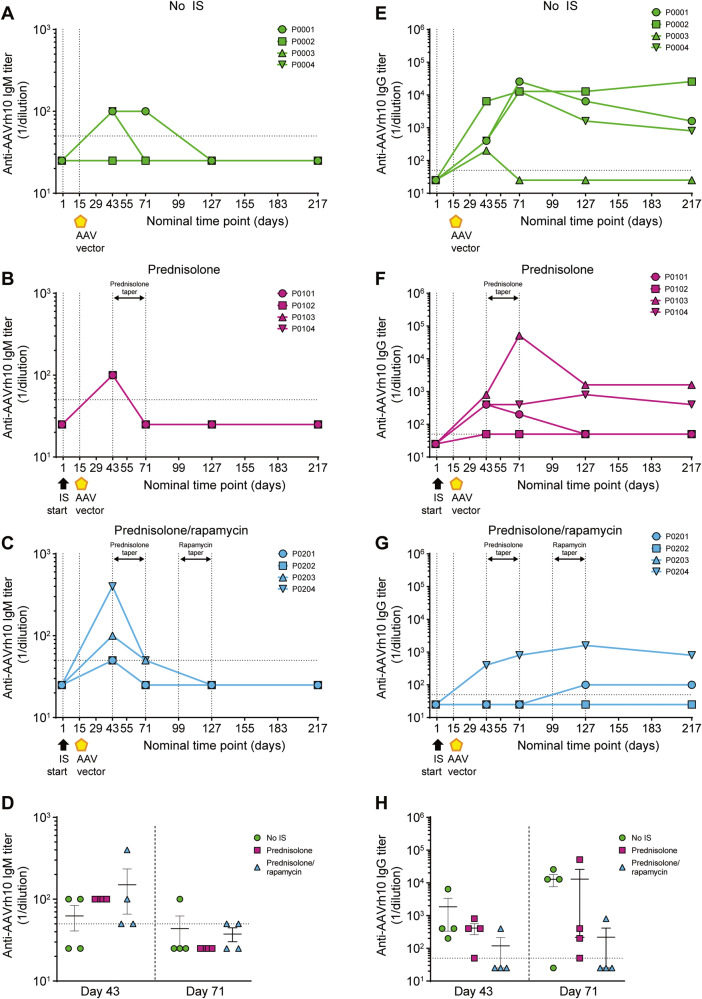
Prednisolone/rapamycin combination reduces mean anti-AAVrh10 IgG but has no impact on anti-AAVrh10 IgM. Serum anti-AAVrh10 IgM and anti-AAVrh10 IgG titers were evaluated in male cynomolgus macaques by ELISA. **A**–**C** anti-AAVrh10 IgM and **E**–**G** anti-AAVrh10 IgG titers are shown for each individual cynomolgus macaque at all time points analyzed. **D** anti-AAVrh10 IgM and **H** anti-AAVrh10 IgG titers at days 43 and 71. Data are represented as mean titer per group ± SEM. Horizontal dashed lines at anti-AAVrh10 IgM/IgG titer (1/dilution) = 50 indicate assay limit of detection; samples below limit of detection are plotted as half the assay limit. Vertical dashed lines in **A**–**C** and **E**–**G** at days 1 and 15 indicate start of IS regimen and AAV administration, respectively. Vertical dashed lines in **A**–**C** and **E**–**G** at days 43, 71, 99, and 127 indicate tapering of IS regimens. Statistical analysis in **D** and **H**, days 43 and 71, was performed using Kruskal–Wallis test with Dunn’s multiple comparisons test; all values calculated were *p* ≥ 0.05. AAV adeno-associated virus, IS immunosuppression.

To further characterize the reduced anti-AAVrh10 NAb response observed in the prednisolone/rapamycin group, the Ig class of anti-AAVrh10 antibodies was assessed at select time points (Fig. 7). Animal P0201 exhibited transient anti-AAVrh10 IgM, with the emergence of anti-AAVrh10 IgG after rapamycin taper (Fig. 7C, G). There was no apparent anti-AAVrh10 IgG development in animals P0202 and P0203; both animals exhibited transient anti-AAVrh10 IgM (Fig. 7C, G). In contrast, animal P0204 displayed an increased anti-AAVrh10 IgG NAb titer at day 43, which was sustained through to the end of the study (Fig. 7G, H). Total IgM (Supplementary Fig. S11A) and IgG (Supplementary Fig. S11B) were also evaluated as controls, and there was no apparent change over time. Overall, these results suggest that prophylactic prednisolone/rapamycin may inhibit the development of antigen-specific anti-AAV capsid NAbs by reducing IgM to IgG isotype switching.

#### IFN and PC gene signature characterization

A panel of 20 genes was used to characterize the IFN response after AAV vector administration in study 2 (Supplementary Fig. S12, Supplementary Table S2) [77]. Analysis of this IFN signature showed an approximately two- to five-fold increase in half of the study animals 24 h after gene therapy, with greater longitudinal fluctuation observed in the no IS group (Supplementary Fig. S12A–D). No additional notable trends were associated with a particular group. Furthermore, unbiased clustering of individual gene expression levels revealed a lack of group association (Supplementary Fig. S12E).

**Fig. 8:**
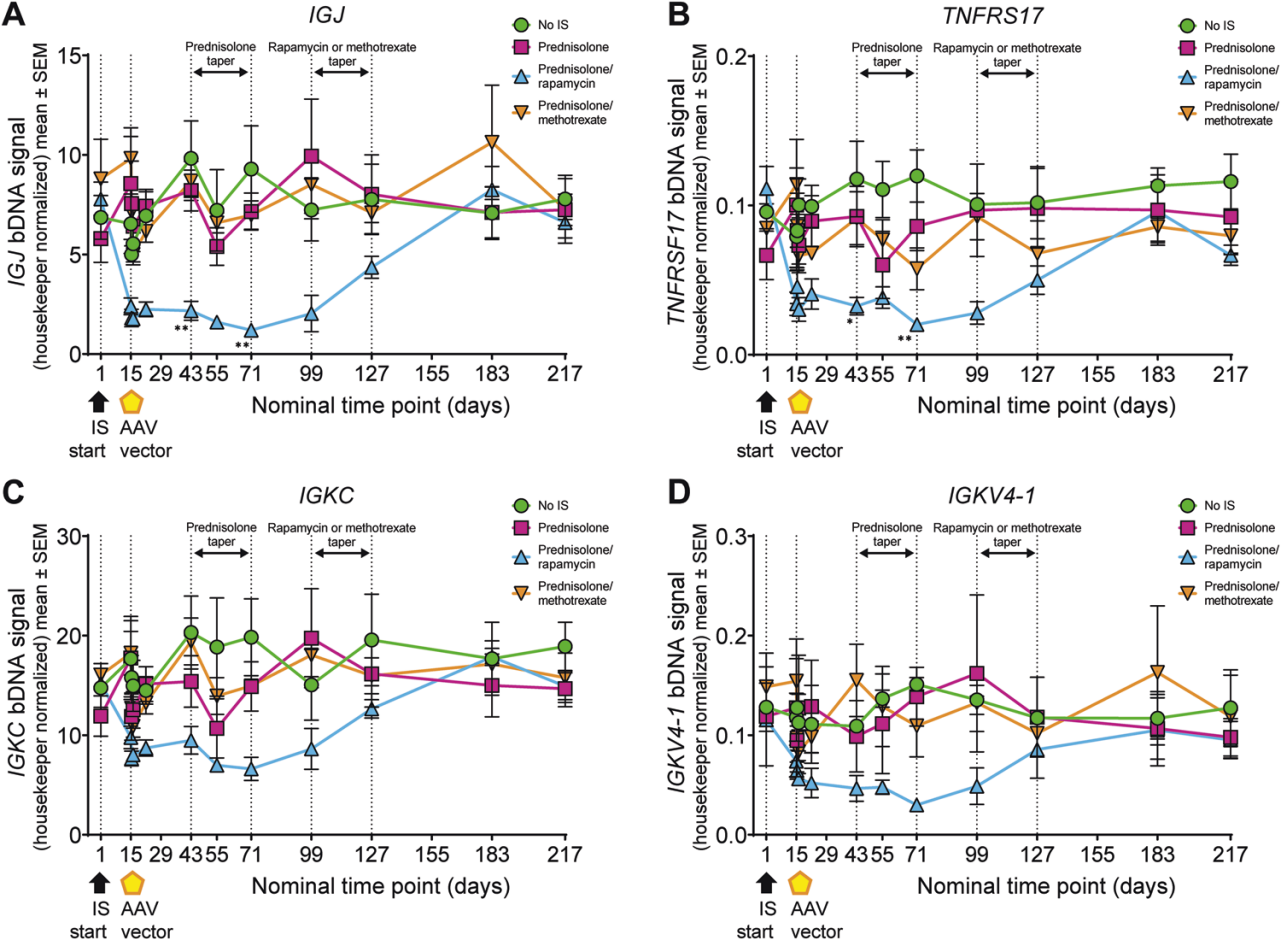
The PC gene signature is significantly reduced by the prednisolone/rapamycin combination. The PC gene signature was evaluated in individual male cynomolgus macaques by determining the expression of five genes in whole blood using a bDNA assay at the indicated time points. Shown are bDNA signals normalized to expression of housekeeping genes for **A** *IGJ*, **B** *TNFRSF17*, **C** *IGKC*, and **D** *IGKV4-1*. Vertical dashed line at days 1 and 15 indicate start of IS regimen and AAV administration, respectively. Vertical dashed lines at days 43, 71, 99, and 127 indicate tapering of IS regimens. Data are represented as mean value per group ± SEM. Statistical analysis in **A**–**D** was performed between the no IS and prednisolone/rapamycin groups using ordinary one-way ANOVA with Tukey’s multiple comparisons test. *p < 0.05; **p < 0.01. See also Supplementary Fig. S13. AAV adeno-associated virus, bDNA branched DNA, IS immunosuppression, PC plasma cell.

The PC gene signature was evaluated in each treatment group using a panel of five genes (Fig. 8, Supplementary Fig. S13) [78]. Reduced expression of *IGJ*, *TNFRSF17*, *IGKC*, and *IGKV4-1* was observed for the prednisolone/rapamycin group before gene therapy administration compared with the no IS group and other IS regimens (Fig. 8A–D). Moreover, *IGJ* and *TNFRSF17* expression was significantly reduced in the prednisolone/rapamycin group compared with no IS at both days 43 and 71 (*IGJ*: days 43 and 71, *p* < 0.01; *TNFRSF17*: day 43, *p* < 0.05; day 71, *p* < 0.01) (Fig. 8A, B). A similar kinetic of reduction and return was observed for *IGHA* for the one animal in this group with elevated levels at baseline (data not shown). The reduced expression of *IGJ*, *TNFRSF17*, *IGKC*, and *IGKV4-1* in prednisolone/rapamycin-treated animals was apparent following IS and prior to vector administration and remained low until rapamycin was tapered. Because this reduced gene expression was not observed in the prednisolone-only group, it suggests a selective ability of rapamycin to reduce the PC gene signature.

#### Analysis of rapamycin and prednisolone on PC differentiation and antibody production in vitro

To further explore the ability of prednisolone and rapamycin to modulate PCs, healthy human PBMCs were stimulated with CpG and IL-2 to differentiate PCs from B cells in vitro (Supplementary Fig. S14). Compared with no IS, rapamycin treatment alone significantly reduced the frequency of CD19+ IgD− CD27+ CD38+ PCs and significantly increased the proportion of FSC^lo^SSC^hi^ apoptotic PCs (Supplementary Fig. S14A, B). Furthermore, a trend for a reduction in the proportion of dividing CD19+ cells, as measured by CFSE dilution, was observed after rapamycin treatment (Supplementary Fig. S14C). Prednisolone alone or in combination with rapamycin did not further influence these parameters. mTOR signaling was analyzed by evaluation of pS6 (Ser240) and XBP-1S expression. In differentiating CD19+ cells after treatment with rapamycin, there was a trend for reduced pS6, and XBP-1S was significantly reduced; there was no additional effect observed with prednisolone alone or in combination with rapamycin (Supplementary Fig. S14D, E). Additionally, rapamycin treatment prevented the production of IgM at 10 and 100 ng/mL and the production of IgG at 10 ng/mL, as measured in the culture supernatant; prednisolone had no observed effect on antibody production (Supplementary Fig. S14F, G).

## Discussion

Significant progress has been made in the treatment of rare diseases with AAV vector gene therapy; however, host immune responses remain an important limiting factor and compromise therapeutic outcomes. The aim of the current report was to address this issue by investigating novel combinations of prophylactic IS regimens in two cynomolgus macaque studies.

The first study compared no IS with prednisolone, rapamycin, rapamycin and cyclosporin A in combination, or cyclosporin A and azathioprine in combination. Overall, delivery of AAVrh10.EnTTR.TTR.hFVIIIco-SQ.75 resulted in detection of plasma hFVIII expression in all groups irrespective of treatment, suggesting that AAV vector transduction and expression occur regardless of the IS regimen. The greatest magnitude of plasma hFVIII expression was observed in the prednisolone group, and therefore, prophylactic prednisolone treatment may provide benefit to vector gene expression. However, longitudinal measurements of circulating hFVIII were confounded by anti-FVIII antibodies; therefore, in-life liver biopsies of vector DNA and mRNA may be useful for future studies to assess durability with or without IS [37].

The incidence of anti-hFVIII IgG was lower than expected in the no IS group, based on a previous study in which all macaques administered an AAVrh10.hFVIII vector developed anti-hFVIII antibodies [62]. In the current report, the vector dose was slightly lower and may be responsible for the lower observed incidence of anti-hFVIII antibodies. Alternatively, heterogeneity in response to AAV vector gene therapy in macaques may also play a role. Of animals that developed anti-hFVIII IgG in study 1 of the current report, reductions in hFVIII plasma levels correlated with anti-hFVIII IgG development, suggesting that the production of anti-hFVIII IgG blocks transgene detection and/or reduces expression of the transduced transgene. Gene therapy eligibility for hFVIII and hFIX currently requires long-term recombinant hFVIII/hFIX use without the development of anti-factor antibodies, which may lead to preselection of tolerant individuals. In the current report, there is a theoretical risk that increased transgene expression as a result of IS could potentially lead to an increased risk for anti-factor antibody development. However, it is important to note that anti-hFVIII IgG was observed in the no IS animals of study 2. The anti-hFVIII IgG results from study 1 should be interpreted with caution, given that group sizes were small and NHP studies are inherently heterogenous.

In the first study, all animals developed anti-AAVrh10 NAbs after vector administration, regardless of treatment group. This finding was expected given the high capsid content administered and is consistent with previous studies [29, 30, 33, 82]. Unexpectedly, three out of four of the azathioprine/cyclosporin A–treated animals in study 1 demonstrated an increase in anti-AAVrh10 NAb titers before vector administration, although they exhibited low titers at screening. Analysis of immunosuppressant dose levels demonstrated toxicity in the in vitro NAb assay at the upper limits only, and the assay drug tolerance is well above the expected IS exposure in vivo. Therefore, it is unlikely that the observed increase in anti-AAVrh10 NAb titers between screening and vector dosing were in vitro artifacts. Rather, the IS regimens may have altered antibody production before administration of the vector, or the animals may have seroconverted due to unplanned natural exposure. The three animals in the azathioprine/cyclosporin A group that developed anti-AAVrh10 NAbs before vector administration showed reduced hFVIII expression, liver vector DNA levels, and hFVIII transcripts when compared with the one animal in this group whose anti-AAVrh10 NAb titers remained below the LOD. Therefore, evaluation of whether anti-AAV NAb development before AAV gene therapy is a result of prophylactic IS or natural seroconversion, and the potential impact on gene transfer, is warranted in future studies. In study 1 of this report, NHPs treated with azathioprine/cyclosporin A demonstrated a decrease in switching from anti-AAVrh10 IgM to IgG after vector administration, suggesting that this regimen may reduce Ig class switching. These data are compelling, given previous reports describing decreased anti-capsid IgG development with IS agents after AAV gene therapy administration, including rapamycin encapsulated in synthetic vaccine particles, and mycophenolate mofetil and rapamycin in combination [63, 83].

In Study 1, a visual relationship between liver vector DNA, liver hFVIII mRNA, blood hFVIII and liver hFVIII ISH hybridization signal suggest that AAVrh10.EnTTR.TTR.hFVIIIco-SQ.PA75 is sustained in the liver and effective through 169 days. The exception to this finding is for animals P0401 and P0402, where anti-hFVIII antibodies were observed in the peripheral blood. We hypothesize that anti-hFVIII antibodies interfered with the assay to detect hFVIII. It is interesting to speculate that these antibodies may also interfere with hFVIII activity, although this has not been directly tested. However, if anti-hFVIII antibodies limit the ability of hFVIII to be detected and active, pharmacologic removal of these antibodies with agents such as the B cell depleting combination of rituximab (anti-CD20) and bortezomib (proteasome inhibitor) or IdeS, may restore gene therapy-driven hFVIII.

It is also interesting to note in Study 1, by ISH, that animals P0401 and P0403 exhibited higher puncta per cell, with more cells with an ISH score of 3 or 4 (between 50–99 and >100 puncta/cell, respectively), as compared with the other study animals (Supplementary Fig. S7C). Even within the same treatment group, animals P0401 and P0403 had lower levels of vector DNA (Supplementary Fig. S7D) and similar levels of hFVIII mRNA (Supplementary Fig. S7E), compared with animal P0402. Likewise, several animals had numerous cells with an ISH score of 1 (1–9 puncta/cell) and lower corresponding hFVIII expression. Further study of the molecular and cellular mechanisms leading to very low or very high individual cell expression is warranted.

In the second study presented in the current report, we further explored prednisolone IS treatment alone or in combination with rapamycin or methotrexate, given that prophylactic prednisolone resulted in the highest level of plasma hFVIII expression in study 1. In addition, considering the study 1 observation of GI issues in NHPs that received rapamycin alone or rapamycin/cyclosporin A that resulted in early euthanasia, additional procedures were added for study 2. Recrudescence of a low-level preexisting infection may have been the cause for this loss; therefore, for the second study, additional pathogen screening was performed, animals were treated prophylactically with fenbendazole, and rapamycin blood levels were monitored to achieve a target range that was expected to be well tolerated. All three IS regimens were well tolerated, and hFVIII expression was observed in all four study groups. The animals receiving prednisolone/rapamycin showed the greatest increase in hFVIII expression in the blood, and in fact, one animal exhibited sustained hFVIII expression throughout the study. This suggests that prednisolone/rapamycin may provide benefit to sustained vector gene expression and may induce immune tolerance to AAV gene therapy in a subset of animals. Unexpectedly, hFVIII expression observed for the prednisolone group in study 2 was not as high as that in study 1, suggesting animal heterogeneity of response, or lab-to-lab variations in vector analytics.

In study 2, delayed anti-hFVIII antibody development was observed in a subset of animals from each IS treatment group when compared with no IS, suggesting that prednisolone alone or in combination with rapamycin or methotrexate may delay the production of anti-FVIII antibodies. Additionally, animals treated with prednisolone/methotrexate showed a reduction in the magnitude of the response, in agreement with several publications documenting reduced anti–α-glucosidase antibodies with methotrexate in enzyme replacement therapy for Pompe disease [84–87]. Across treatment arms, the lowest anti-AAVrh10 NAb titers were observed with prednisolone/rapamycin, suggesting that this IS combination may be useful for reducing AAVrh10 NAb development and blocking switching from IgM to IgG. However, one animal in the prednisolone/rapamycin group demonstrated a rapid and high titer of anti-AAVrh10 NAbs and anti-AAVrh10 IgG (P0204), suggestive of either preexisting cross-reactive immunological memory, insufficiently high rapamycin levels, or other unknown factors. Although it is not feasible to test all possible cross-reactive antigens, baseline anti-AAV8 (a capsid from the same clade as AAVrh10 [88]) NAb levels in this animal were tested and were below the detection limit of the assay. Overall, these outcomes suggest that prednisolone/rapamycin may reduce the development of anti-transgene IgG and anti-capsid NAbs.

Historically, prednisolone has been used broadly in the clinic for AAV gene therapy to mitigate immune responses after vector administration [8, 10, 11, 56], but has been less well studied in NHPs. In a study of rAAVrh74-delivered *GALGT2* gene therapy to skeletal muscle in rhesus macaques, prophylactic prednisolone or prednisolone in combination with tacrolimus and mycophenolate mofetil showed a trend for reduced anti-vector antibodies; however, neither approach significantly affected *GALGT2* expression [89]. A later study from the same group assessed the impact of preexisting anti-vector antibodies on transduction. Here, prophylactic prednisone significantly reduced CD8+ T-cell infiltrates compared with no IS cohorts. A trend for reduced IFN-γ–positive T cells and serum anti-rAAVrh74 antibodies was also observed but was not significant, possibly due to small group sizes [90]. In a recent cynomolgus macaque study of DTX301 (avalotcagene ontaparvovec) gene therapy, prednisolone-treated animals exhibited a trend toward greater vector genome and transgene expression, and a significantly decreased hepatic IFN gene signature compared with no IS; however, these observations waned upon prednisolone withdrawal [37].

Rapamycin is well studied in preclinical models of AAV vector gene therapy and can inhibit AAV vector immunity in part by selectively favoring the expansion and conversion of regulatory T cells over effector T cells [59, 91–94]. Coadministration of AAV vectors with nanoparticles containing rapamycin in mice robustly controlled capsid immunogenicity [63]. Importantly, the tolerogenic rapamycin particles allowed successful readministration of vector in both mice and NHPs. A single case study demonstrated promising results for the clinical use of rapamycin in combination with rituximab in AAV1-mediated gene therapy in a child with Pompe disease [61]. This IS regimen is currently being applied prophylactically in a clinical trial of AAV9 gene therapy in patients with Pompe disease [60].

In study 2 of the current analysis, the prednisolone/rapamycin combination significantly reduced the PC gene signature during the rapamycin dosing window in comparison with the other treatment arms. In agreement with this finding, a high-throughput screen of compounds for ability to inhibit antibody production reported that rapamycin inhibited B-cell activation and plasmablast formation [79]. In a mouse model of preexisting AAV9 antibodies, the combination of prednisolone and rapamycin was the most effective at reducing serum anti-capsid antibodies, in part by reducing the frequency of PCs [95]. The study investigated reversal of preexisting anti-capsid responses in mice, representing important differences from our study; however, the observed effects of combined prednisolone and rapamycin on PCs is interesting to note. Finally, we have shown in this report that rapamycin treatment inhibited B-cell and PC function in vitro, corroborating previous reports demonstrating the mechanistic importance of mTOR in B-cell function [96–99]. Taken together, these results support our findings that prednisolone/rapamycin may provide therapeutic benefit for AAV-mediated gene therapy by reducing the development and function of PCs.

The current report is the first demonstration in gene therapy of the in vivo effect of rapamycin on PC gene signature with concordant improvement of gene therapy outcomes in cynomolgus macaques. Limitations of the current analysis include the small sample sizes and the rapamycin-related health issues in study 1 that led to the therapeutic monitoring of exposure in study 2. Analysis of the IS regimens explored here in additional gene therapy models that include both males and females is also warranted.

Together, the results of the two studies in this report present a careful and thorough analysis of IS regimens in a preclinical model of AAVrh10.hFVIII gene therapy. The combination of prednisolone and rapamycin was identified as a prophylactic IS regimen that may improve therapeutic outcomes, including possible enhanced transgene expression, reduction of anti-AAVrh10 NAb development and IgG class switching, and decreased PC gene signature. Therefore, this regimen has the potential for translation to clinical studies in humans. Future studies will benefit from an increased number of animals and an investigation into timing and dose of prednisolone/rapamycin with or without additional IS to prolong observed therapeutic benefits to gene therapy.

### Supplementary information

Supplemental material

## Data Availability

The data that support the reported results are available from the corresponding authors upon reasonable request.

## References

[1] Bainbridge JWB, Mehat MS, Sundaram V, Robbie SJ, Barker SE, Ripamonti C (2015). Long-term effect of gene therapy on Leber’s congenital amaurosis. N Engl J Med.

[2] Binks M. Pfizer gene therapy (PF-06939926) in Duchenne muscular dystrophy. In: Workshop on Systemic Immunogenicity Considerations for AAV-mediated Gene Therapy (National Center for Advancing Translational Sciences); November 30–December 1. Virtual; 2021.

[3] Gaudet D, Méthot J, Déry S, Brisson D, Essiembre C, Tremblay G (2013). Efficacy and long-term safety of alipogene tiparvovec (AAV1-LPLS^447X^) gene therapy for lipoprotein lipase deficiency: an open-label trial. Gene Ther.

[4] George LA, Sullivan SK, Giermasz A, Rasko JEJ, Samelson-Jones BJ, Ducore J (2017). Hemophilia B gene therapy with a high-specific-activity factor IX variant. N Engl J Med.

[5] Kishnani PS, Sun B, Koeberl DD (2019). Gene therapy for glycogen storage diseases. Hum Mol Genet.

[6] Le Guiner C, Servais L, Montus M, Larcher T, Fraysse B, Moullec S (2017). Long-term microdystrophin gene therapy is effective in a canine model of Duchenne muscular dystrophy. Nat Commun.

[7] Maguire AM, Russell S, Wellman JA, Chung DC, Yu Z-F, Tillman A (2019). Efficacy, safety, and durability of voretigene neparvovec-rzyl in *RPE65* mutation–associated inherited retinal dystrophy: results of phase 1 and 3 trials. Ophthalmology.

[8] Mendell JR, Al-Zaidy S, Shell R, Arnold WD, Rodino-Klapac LR, Prior TW (2017). Single-dose gene-replacement therapy for spinal muscular atrophy. N Engl J Med.

[9] Murillo O, Moreno D, Gazquez C, Barberia M, Cenzano I, Navarro I (2019). Liver expression of a MiniATP7B gene results in long-term restoration of copper homeostasis in a Wilson disease model in mice. Hepatology.

[10] Nathwani AC, Reiss UM, Tuddenham EG, Rosales C, Chowdary P, McIntosh J (2014). Long-term safety and efficacy of factor IX gene therapy in hemophilia B. N Engl J Med.

[11] Pasi KJ, Rangarajan S, Mitchell N, Lester W, Symington E, Madan B (2020). Multiyear follow-up of AAV5-hFVIII-SQ gene therapy for hemophilia A. N Engl J Med.

[12] Raper SE, Yudkoff M, Chirmule N, Gao G-P, Nunes F, Haskal ZJ (2002). A pilot study of in vivo liver-directed gene transfer with an adenoviral vector in partial ornithine transcarbamylase deficiency. Hum Gene Ther.

[13] Smith BK, Collins SW, Conlon TJ, Mah CS, Lawson LA, Martin AD (2013). Phase I/II trial of adeno-associated virus-mediated alpha-glucosidase gene therapy to the diaphragm for chronic respiratory failure in Pompe disease: initial safety and ventilatory outcomes. Hum Gene Ther.

[14] Tan W-H. AAV8 gene therapy as a potential treatment in adults with late-onset OTC deficiency: results from a phase 1/2 clinical trial. The American Society of Gene & Cell Therapy 23rd Annual Meeting; 12–15 May 2020. Virtual.

[15] Wang D, Tai PWL, Gao G (2019). Adeno-associated virus vector as a platform for gene therapy delivery. Nat Rev Drug Discov.

[16] Hoy SM (2019). Onasemnogene abeparvovec: first global approval. Drugs.

[17] Ylä-Herttuala S (2012). Endgame: Glybera finally recommended for approval as the first gene therapy drug in the European Union. Mol Ther.

[18] Heo YA (2023). Etranacogene Dezaparvovec: first approval. Drugs.

[19] Blair HA (2022). Valoctocogene Roxaparvovec: first approval. Drugs.

[20] Ronzitti G, Gross D-A, Mingozzi F (2020). Human immune responses to adeno-associated virus (AAV) vectors. Front Immunol.

[21] Verdera HC, Kuranda K, Mingozzi F (2020). AAV vector immunogenicity in humans: a long journey to successful gene transfer. Mol Ther.

[22] Boutin S, Monteilhet V, Veron P, Leborgne C, Benveniste O, Montus MF (2010). Prevalence of serum IgG and neutralizing factors against adeno-associated virus (AAV) types 1, 2, 5, 6, 8, and 9 in the healthy population: implications for gene therapy using AAV vectors. Hum Gene Ther.

[23] Calcedo R, Vandenberghe LH, Gao G, Lin J, Wilson JM (2009). Worldwide epidemiology of neutralizing antibodies to adeno-associated viruses. J Infect Dis.

[24] Calcedo R, Wilson JM (2016). AAV natural infection induces broad cross-neutralizing antibody responses to multiple AAV serotypes in chimpanzees. Hum Gene Ther Clin Dev.

[25] Erles K, Sebökovà P, Schlehofer JR (1999). Update on the prevalence of serum antibodies (IgG and IgM) to adeno-associated virus (AAV). J Med Virol.

[26] Perocheau DP, Cunningham S, Lee J, Antinao Diaz J, Waddington SN, Gilmour K (2019). Age-related seroprevalence of antibodies against AAV-LK03 in a UK population cohort. Hum Gene Ther.

[27] Wang L, Calcedo R, Wang H, Bell P, Grant R, Vandenberghe LH (2010). The pleiotropic effects of natural AAV infections on liver-directed gene transfer in macaques. Mol Ther.

[28] Klamroth R, Hayes G, Andreeva T, Gregg K, Suzuki T, Mitha IH (2022). Global seroprevalence of pre-existing immunity against AAV5 and other AAV serotypes in people with hemophilia A. Hum Gene Ther.

[29] Jiang H, Couto LB, Patarroyo-White S, Liu T, Nagy D, Vargas JA (2006). Effects of transient immunosuppression on adenoassociated, virus-mediated, liver-directed gene transfer in rhesus macaques and implications for human gene therapy. Blood.

[30] Long BR, Sandza K, Holcomb J, Crockett L, Hayes GM, Arens J (2019). The impact of pre-existing immunity on the non-clinical pharmacodynamics of AAV5-based gene therapy. Mol Ther Methods Clin Dev.

[31] Manno CS, Pierce GF, Arruda VR, Glader B, Ragni M, Rasko JJ (2006). Successful transduction of liver in hemophilia by AAV-factor IX and limitations imposed by the host immune response. Nat Med.

[32] Scallan CD, Jiang H, Liu T, Patarroyo-White S, Sommer JM, Zhou S (2006). Human immunoglobulin inhibits liver transduction by AAV vectors at low AAV2 neutralizing titers in SCID mice. Blood.

[33] Wang L, Calcedo R, Bell P, Lin J, Grant RL, Siegel DL (2011). Impact of pre-existing immunity on gene transfer to nonhuman primate liver with adeno-associated virus 8 vectors. Hum Gene Ther.

[34] Kuranda K, Jean-Alphonse P, Leborgne C, Hardet R, Collaud F, Marmier S (2018). Exposure to wild-type AAV drives distinct capsid immunity profiles in humans. J Clin Invest.

[35] Mingozzi F, Maus MV, Hui DJ, Sabatino DE, Murphy SL, Rasko JE (2007). CD8^+^ T-cell responses to adeno-associated virus capsid in humans. Nat Med.

[36] Nathwani AC, Rosales C, McIntosh J, Rastegarlari G, Nathwani D, Raj D (2011). Long-term safety and efficacy following systemic administration of a self-complementary AAV vector encoding human FIX pseudotyped with serotype 5 and 8 capsid proteins. Mol Ther.

[37] Wang L, Warzecha CC, Kistner A, Chichester JA, Bell P, Buza EL (2022). Prednisolone reduces the interferon response to AAV in cynomolgus macaques and may increase liver gene expression. Mol Ther Methods Clin Dev.

[38] Calcedo R, Somanathan S, Qin Q, Betts MR, Rech AJ, Vonderheide RH (2017). Class I-restricted T-cell responses to a polymorphic peptide in a gene therapy clinical trial for α-1-antitrypsin deficiency. Proc Natl Acad Sci USA.

[39] Mendell JR, Campbell K, Rodino-Klapac L, Sahenk Z, Shilling C, Lewis S (2010). Dystrophin immunity in Duchenne’s muscular dystrophy. N Engl J Med.

[40] Tardieu M, Zérah M, Gougeon M-L, Ausseil J, de Bournonville S, Husson B (2017). Intracerebral gene therapy in children with mucopolysaccharidosis type IIIB syndrome: an uncontrolled phase 1/2 clinical trial. Lancet Neurol.

[41] Ciesielska A, Hadaczek P, Mittermeyer G, Zhou S, Wright JF, Bankiewicz KS (2013). Cerebral infusion of AAV9 vector-encoding non-self proteins can elicit cell-mediated immune responses. Mol Ther.

[42] Limberis MP, Figueredo J, Calcedo R, Wilson JM (2007). Activation of CFTR-specific T cells in cystic fibrosis mice following gene transfer. Mol Ther.

[43] Hösel M, Broxtermann M, Janicki H, Esser K, Arzberger S, Hartmann P (2012). Toll-like receptor 2–mediated innate immune response in human nonparenchymal liver cells toward adeno-associated viral vectors. Hepatology.

[44] Martino AT, Suzuki M, Markusic DM, Zolotukhin I, Ryals RC, Moghimi B (2011). The genome of self-complementary adeno-associated viral vectors increases toll-like receptor 9-dependent innate immune responses in the liver. Blood.

[45] Rogers GL, Shirley JL, Zolotukhin I, Kumar SRP, Sherman A, Perrin GQ (2017). Plasmacytoid and conventional dendritic cells cooperate in crosspriming AAV capsid-specific CD8^+^ T cells. Blood.

[46] Shirley JL, Keeler GD, Sherman A, Zolotukhin I, Markusic DM, Hoffman BE (2020). Type I IFN sensing by cDCs and CD4^+^ T cell help are both requisite for cross-priming of AAV capsid-specific CD8^+^ T cells. Mol Ther.

[47] Zhang X. The effect of innate immune response MAVS sensor on AAV long-term transduction. The American Society of Gene & Cell Therapy 23rd Annual Meeting; 12–15 May 2020. Virtual.

[48] Zhu J, Huang X, Yang Y (2009). The TLR9-MyD88 pathway is critical for adaptive immune responses to adeno-associated virus gene therapy vectors in mice. J Clin Invest.

[49] Bertin B, Veron P, Leborgne C, Deschamps J-Y, Moullec S, Fromes Y (2020). Capsid-specific removal of circulating antibodies to adeno-associated virus vectors. Sci Rep.

[50] Leborgne C, Barbon E, Alexander JM, Hanby H, Delignat S, Cohen DM (2020). IgG-cleaving endopeptidase enables in vivo gene therapy in the presence of anti-AAV neutralizing antibodies. Nat Med.

[51] Potter R, Khan S, Snedeker J, Adegboye K, Haile A, Sayanjali B, et al. The safety and efficacy of pre-treatment with imlifidase prior to adeno associated virus (AAV)-based gene therapy in non-human primates with pre-existing anti-AAVrh74 antibodies. The 26th Annual Meeting of the American Society of Gene & Cell Therapy; 16–20 May 2023. Los Angeles, CA, USA.

[52] Chandler LC, Barnard AR, Caddy SL, Patrício MI, McClements ME, Fu H (2019). Enhancement of adeno-associated virus-mediated gene therapy using hydroxychloroquine in murine and human tissues. Mol Ther Methods Clin Dev.

[53] Faust SM. CpG-motifs within AAV vectors triggers immune activation upon hepatic gene transfer. The American Society of Gene & Cell Therapy 23rd Annual Meeting; 12–15 May 2002. Virtual.

[54] Faust SM, Bell P, Cutler BJ, Ashley SN, Zhu Y, Rabinowitz JE (2013). CpG-depleted adeno-associated virus vectors evade immune detection. J Clin Invest.

[55] Li C, He Y, Nicolson S, Hirsch M, Weinberg MS, Zhang P (2013). Adeno-associated virus capsid antigen presentation is dependent on endosomal escape. J Clin Invest.

[56] Russell S, Bennett J, Wellman JA, Chung DC, Yu Z-F, Tillman A (2017). Efficacy and safety of voretigene neparvovec (AAV2-hRPE65v2) in patients with *RPE65*-mediated inherited retinal dystrophy: a randomised, controlled, open-label, phase 3 trial. Lancet.

[57] Konkle BA, Walsh CE, Escobar MA, Josephson NC, Young G, von Drygalski A (2021). BAX 335 hemophilia B gene therapy clinical trial results: potential impact of CpG sequences on gene expression. Blood.

[58] George LA, Monahan PE, Eyster ME, Sullivan SK, Ragni MV, Croteau SE (2021). Multiyear factor VIII expression after AAV gene transfer for hemophilia A. N Engl J Med.

[59] Biswas M, Rogers GL, Sherman A, Byrne BJ, Markusic DM, Jiang H (2017). Combination therapy for inhibitor reversal in haemophilia A using monoclonal anti-CD20 and rapamycin. Thromb Haemost.

[60] Corti M, Cleaver B, Clément N, Conlon TJ, Faris KJ, Wang G (2015). Evaluation of readministration of a recombinant adeno-associated virus vector expressing acid alpha-glucosidase in Pompe disease: preclinical to clinical planning. Hum Gene Ther Clin Dev.

[61] Corti M, Elder M, Falk D, Lawson L, Smith B, Nayak S (2014). B-cell depletion is protective against anti-AAV capsid immune response: a human subject case study. Mol Ther Methods Clin Dev.

[62] Greig JA, Nordin JML, White JW, Wang Q, Bote E, Goode T (2018). Optimized adeno-associated viral-mediated human factor VIII gene therapy in cynomolgus macaques. Hum Gene Ther.

[63] Meliani A, Boisgerault F, Hardet R, Marmier S, Collaud F, Ronzitti G (2018). Antigen-selective modulation of AAV immunogenicity with tolerogenic rapamycin nanoparticles enables successful vector re-administration. Nat Commun.

[64] Erturk-Hasdemir D, Scott W, Kistner A, Cardwell L, Serio J, Sullivan L, et al. Immunosuppression to inhibit capsid-specific humoral immune responses in high-dose AAV gene therapy in cynomolgus macaques. The 26th Annual Meeting of the American Society of Gene & Cell Therapy; 16–20 May 2023; Los Angeles, CA, USA.

[65] Patel K, Cai W, Khatri A, Marie L, Bazile M, Lopez E, et al. Combination immunosuppression prevents toxicity and increases liver transduction in cynomolgus macaques administered with high dose AAV vector. The 26th Annual Meeting of the American Society of Gene & Cell Therapy; 16–20 May 2023; Los Angeles, CA, USA.

[66] Kakkis E, Lester T, Yang R, Tanaka C, Anand V, Lemontt J (2004). Successful induction of immune tolerance to enzyme replacement therapy in canine mucopolysaccharidosis I. Proc Natl Acad Sci USA.

[67] Calcedo R, Chichester JA, Wilson JM (2018). Assessment of humoral, innate, and T-cell immune responses to adeno-associated virus vectors. Hum Gene Ther Methods.

[68] Zimmerman JJ, Harper D, Getsy J, Jusko WJ (2003). Pharmacokinetic interactions between sirolimus and microemulsion cyclosporine when orally administered jointly and 4 h apart in healthy volunteers. J Clin Pharmacol.

[69] Clauss A (1957). [Rapid physiological coagulation method in determination of fibrinogen]. Acta Haematol.

[70] Greig JA, Wang Q, Reicherter AL, Chen S-J, Hanlon AL, Tipper CH (2017). Characterization of adeno-associated viral vector-mediated human factor VIII gene therapy in hemophilia A mice. Hum Gene Ther.

[71] McIntosh J, Lenting PJ, Rosales C, Lee D, Rabbanian S, Raj D (2013). Therapeutic levels of FVIII following a single peripheral vein administration of rAAV vector encoding a novel human factor VIII variant. Blood.

[72] Lock M, Alvira M, Vandenberghe LH, Samanta A, Toelen J, Debyser Z (2010). Rapid, simple, and versatile manufacturing of recombinant adeno-associated viral vectors at scale. Hum Gene Ther.

[73] Lock M, Alvira MR, Chen S-J, Wilson JM (2014). Absolute determination of single-stranded and self-complementary adeno-associated viral vector genome titers by droplet digital PCR. Hum Gene Ther Methods.

[74] Bell P, Moscioni AD, McCarter RJ, Wu D, Gao G, Hoang A (2006). Analysis of tumors arising in male B6C3F1 mice with and without AAV vector delivery to liver. Mol Ther.

[75] Gorovits B, Fiscella M, Havert M, Koren E, Long B, Milton M (2020). Recommendations for the development of cell-based anti-viral vector neutralizing antibody assays. AAPS J.

[76] Meliani A, Leborgne C, Triffault S, Jeanson-Leh L, Veron P, Mingozzi F (2015). Determination of anti-adeno-associated virus vector neutralizing antibody titer with an in vitro reporter system. Hum Gene Ther Methods.

[77] Yao Y, Higgs BW, Morehouse C, de Los Reyes M, Trigona W, Brohawn P (2009). Development of potential pharmacodynamic and diagnostic markers for anti-IFN-α monoclonal antibody trials in systemic lupus erythematosus. Hum Genomics Proteomics.

[78] Streicher K, Morehouse CA, Groves CJ, Rajan B, Pilataxi F, Lehmann KP (2014). The plasma cell signature in autoimmune disease. Arthritis Rheumatol.

[79] Tuijnenburg P, Aan de Kerk DJ, Jansen MH, Morris B, Lieftink C, Beijersbergen RL (2020). High-throughput compound screen reveals mTOR inhibitors as potential therapeutics to reduce (auto)antibody production by human plasma cells. Eur J Immunol.

[80] Böhme D, Beck-Sickinger AG (2015). Controlling toxicity of peptide-drug conjugates by different chemical linker structures. ChemMedChem.

[81] Lamoureux F, Mestre E, Essig M, Sauvage FL, Marquet P, Gastinel LN (2011). Quantitative proteomic analysis of cyclosporine-induced toxicity in a human kidney cell line and comparison with tacrolimus. J Proteomics.

[82] Rangarajan S, Walsh L, Lester W, Perry D, Madan B, Laffan M (2017). AAV5–factor VIII gene transfer in severe hemophilia A. N Engl J Med.

[83] Mingozzi F, Hasbrouck NC, Basner-Tschakarjan E, Edmonson SA, Hui DJ, Sabatino DE (2007). Modulation of tolerance to the transgene product in a nonhuman primate model of AAV-mediated gene transfer to liver. Blood.

[84] Joly MS, Martin RP, Mitra-Kaushik S, Phillips L, D’Angona A, Richards SM (2014). Transient low-dose methotrexate generates B regulatory cells that mediate antigen-specific tolerance to alglucosidase alfa. J Immunol.

[85] Joseph A, Munroe K, Housman M, Garman R, Richards S (2008). Immune tolerance induction to enzyme-replacement therapy by co-administration of short-term, low-dose methotrexate in a murine Pompe disease model. Clin Exp Immunol.

[86] Mendelsohn NJ, Messinger YH, Rosenberg AS, Kishnani PS (2009). Elimination of antibodies to recombinant enzyme in Pompe’s disease. N Engl J Med.

[87] Messinger YH, Mendelsohn NJ, Rhead W, Dimmock D, Hershkovitz E, Champion M (2012). Successful immune tolerance induction to enzyme replacement therapy in CRIM-negative infantile Pompe disease. Genet Med.

[88] Gao G, Vandenberghe LH, Alvira MR, Lu Y, Calcedo R, Zhou X (2004). Clades of Adeno-associated viruses are widely disseminated in human tissues. J Virol.

[89] Chicoine LG, Rodino-Klapac LR, Shao G, Xu R, Bremer WG, Camboni M (2014). Vascular delivery of rAAVrh74.MCK.*GALGT2* to the gastrocnemius muscle of the rhesus macaque stimulates the expression of dystrophin and laminin α2 surrogates. Mol Ther.

[90] Cramer ML, Shao G, Rodino-Klapac LR, Chicoine LG, Martin PT (2017). Induction of T-cell infiltration and programmed death ligand 2 expression by adeno-associated virus in rhesus macaque skeletal muscle and modulation by prednisone. Hum Gene Ther.

[91] Battaglia M, Stabilini A, Roncarolo M-G (2005). Rapamycin selectively expands CD4^+^CD25^+^FoxP3^+^ regulatory T cells. Blood.

[92] Delgoffe GM, Kole TP, Zheng Y, Zarek PE, Matthews KL, Xiao B (2009). The mTOR kinase differentially regulates effector and regulatory T cell lineage commitment. Immunity.

[93] Nayak S, Cao O, Hoffman BE, Cooper M, Zhou S, Atkinson MA (2009). Prophylactic immune tolerance induced by changing the ratio of antigen-specific effector to regulatory T cells. J Thromb Haemost.

[94] Nayak S, Sarkar D, Perrin GQ, Moghimi B, Hoffman BE, Zhou S (2011). Prevention and reversal of antibody responses against factor IX in gene therapy for hemophilia B. Front Microbiol.

[95] Velazquez VM, Meadows AS, Pineda RJ, Camboni M, McCarty DM, Fu H (2017). Effective depletion of pre-existing anti-AAV antibodies requires broad immune targeting. Mol Ther Methods Clin Dev.

[96] Ersching J, Efeyan A, Mesin L, Jacobsen JT, Pasqual G, Grabiner BC (2017). Germinal center selection and affinity maturation require dynamic regulation of mTORC1 kinase. Immunity.

[97] Iwata TN, Ramírez-Komo JA, Park H, Iritani BM (2017). Control of B lymphocyte development and functions by the mTOR signaling pathways. Cytokine Growth Factor Rev.

[98] Raybuck AL, Cho SH, Li J, Rogers MC, Lee K, Williams CL (2018). B cell–intrinsic mTORC1 promotes germinal center–defining transcription factor gene expression, somatic hypermutation, and memory B cell generation in humoral immunity. J Immunol.

[99] Zhang S, Pruitt M, Tran D, Du Bois W, Zhang K, Patel R (2013). B cell–specific deficiencies in mTOR limit humoral immune responses. J Immunol.

