# Supplementary material for: Prednisolone and rapamycin reduce the plasma cell gene signature and may improve AAV gene therapy in cynomolgus macaques
Source: Gene Ther. 2023 Oct 13;31(3-4):128–43. doi: 10.1038/s41434-023-00423-z (PMC10940161; doi:10.1038/s41434-023-00423-z)
Supplement: Supplementary file 1 — Supplemental material [file 41434_2023_423_MOESM1_ESM.pdf]

SUPPLEMENTARY MATERIAL

Prednisolone and rapamycin reduce the plasma cell gene signature and may improve AAV gene therapy in cynomolgus macaques

Alexander Kistner[1], Jessica A. Chichester[2], Lili Wang[2], Roberto Calcedo[2,5], Jenny A. Greig[2], Leah N. Cardwell[3], Margaret C. Wright[1], Julien Couthouis[1], Sunjay Sethi[4], Brian E. McIntosh[6], Kathleen McKeever[1], Samuel Wadsworth[3], James M. Wilson[2], Emil Kakkis[1] and Barbara A. Sullivan[1]

[1]Ultragenyx Pharmaceutical Inc., Novato, CA, USA. [2]Gene Therapy Program, Department of Medicine, Perelman School of Medicine, University of Pennsylvania, Philadelphia, PA, USA. [3]Ultragenyx Gene Therapy, Ultragenyx Pharmaceutical Inc., Cambridge, MA, USA. [4]Charles River Laboratories Inc., Reno, NV, USA. [5]Present address: Affinia Therapeutics, Waltham, MA, USA. [6]Labcorp Drug Development, Madison, WI, USA.

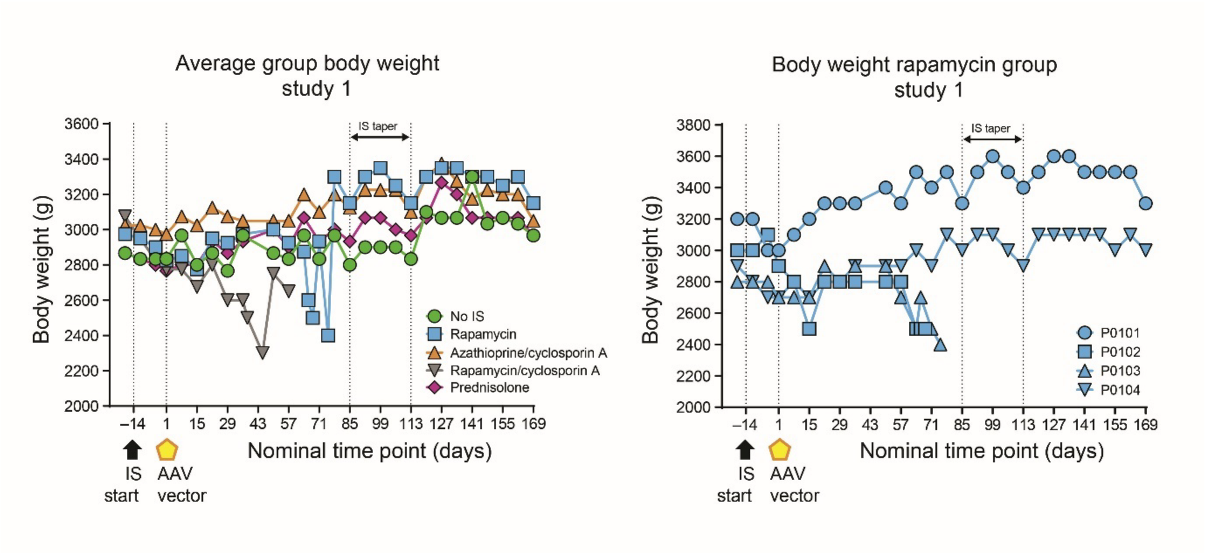

Supplementary Fig. S1. Body weights of study 1 male cynomolgus macaques. Weight in grams was evaluated in individual male cynomolgus macaques at the indicated time points. Left panel: mean weight for the no IS group and for each IS regimen group; scheduled collections are presented only. Right panel: weights for individual cynomolgus macaques from the rapamycin only group; all collection time points are presented. Vertical dashed line at days –14 and 1 indicate start of IS regimen and AAV administration, respectively. Vertical dashed lines at days 85 and 113 indicate IS taper. *AAV* adeno-associated virus, *IS* immunosuppression.

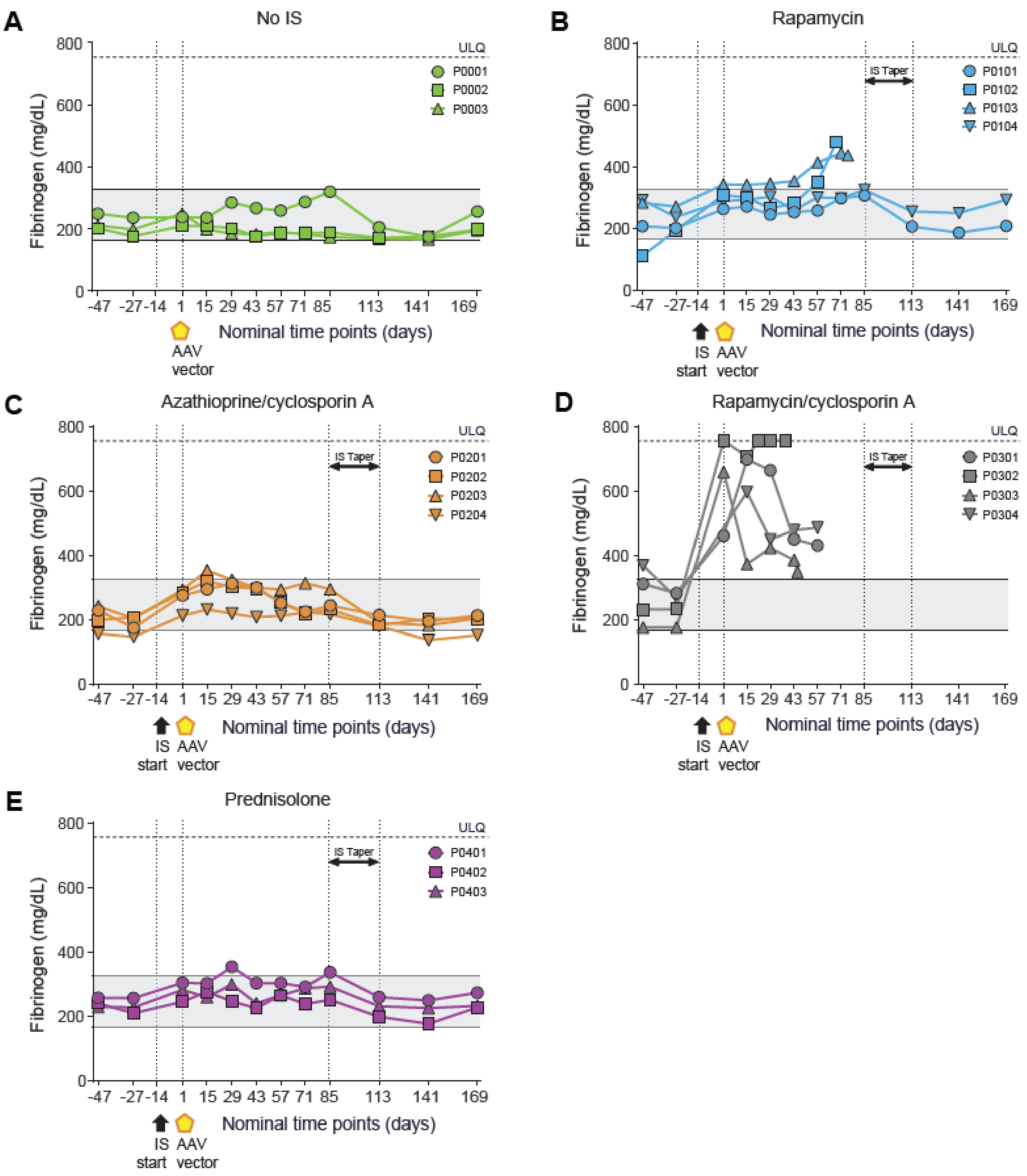

Supplementary Fig. S2. Plasma fibrinogen in study 1. A–E Plasma fibrinogen was evaluated in individual male cynomolgus macaques using the HemosIL® Fibrinogen-C XL assay at the indicated time points. Horizontal dashed lines and gray area indicates reference range. Vertical dashed lines at days –14 and 1 indicate start of IS regimen and AAV administration, respectively. Vertical dashed lines at days 85 and 113 indicate IS taper. *AAV* adeno-associated virus, *IS* immunosuppression, *ULQ*, upper limit of quantitation.

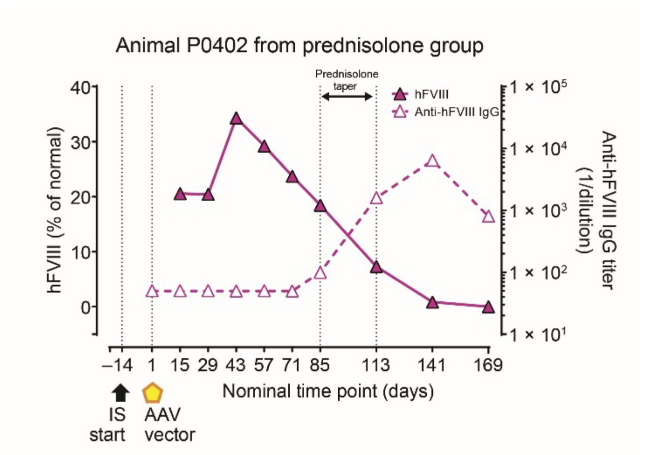

Supplementary Fig. S3. hFVIII plasma expression and anti-hFVIII IgG in animal P0402 from the prednisolone group. Plasma hFVIII levels and serum anti-hFVIII IgG titers were evaluated in individual male cynomolgus macaques by ELISA at the indicated time points. hFVIII (% of normal) and anti-hFVIII IgG titers are shown on the same graph for animal P0402 from the prednisolone group. Vertical dashed lines at days –14 and 1 indicate start of IS regimen and AAV administration, respectively. Vertical dashed lines at days 85 and 113 indicate prednisolone taper. See also Fig. 2. *AAV* adeno-associated virus, *hFVIII* human coagulation factor VIII, *IS* immunosuppression.

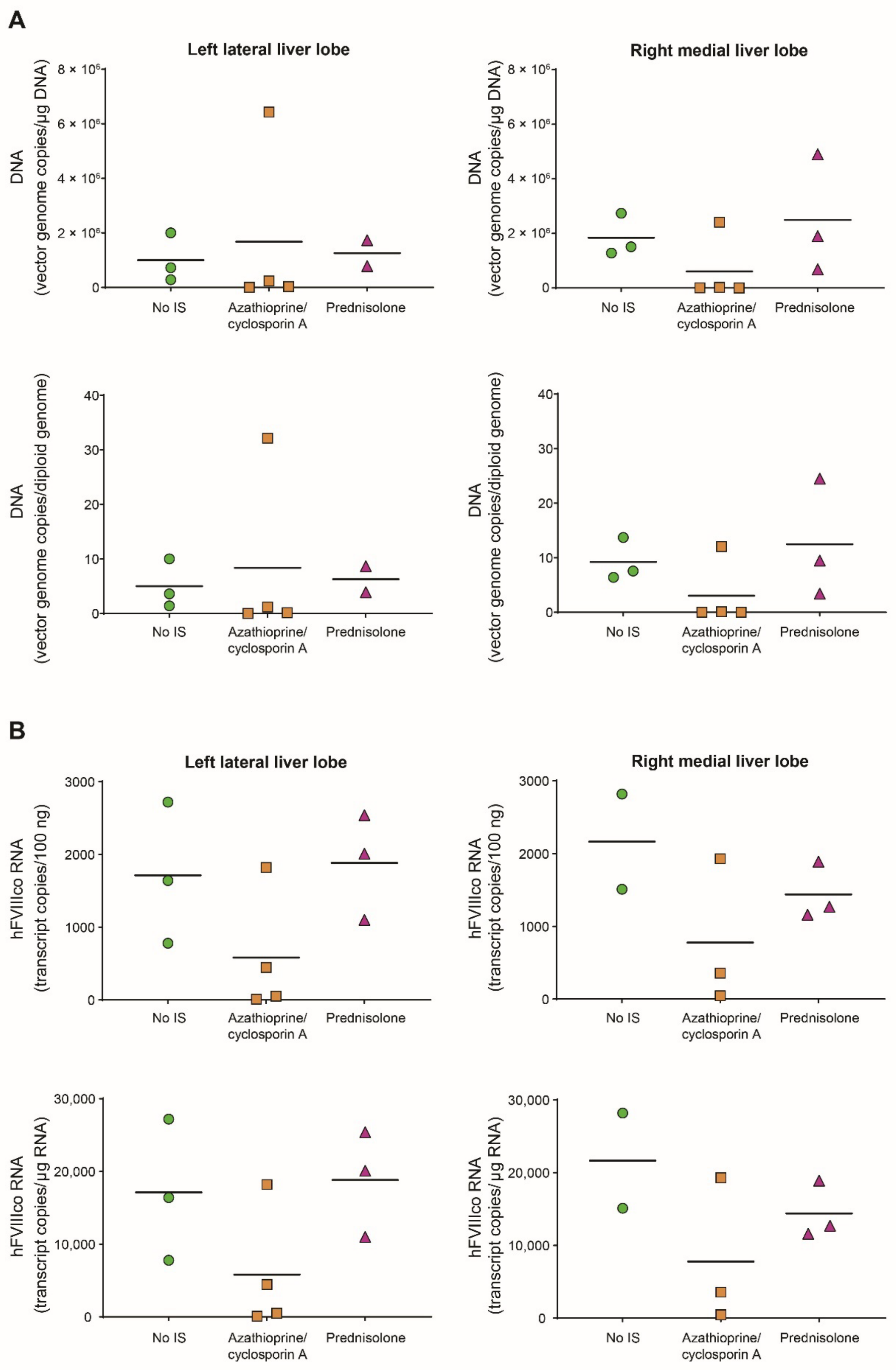

Supplementary Fig. S4. Similar liver biodistribution upon necropsy in the prednisolone group compared with the no IS group. A hFVIII genome copies and **B** hFVIII transcript levels were evaluated in the liver of individual cynomolgus macaques by quantitative PCR (qPCR) or RT-qPCR at day 169 necropsy. *hFVIII* human coagulation factor VIII, *IS* immunosuppression.

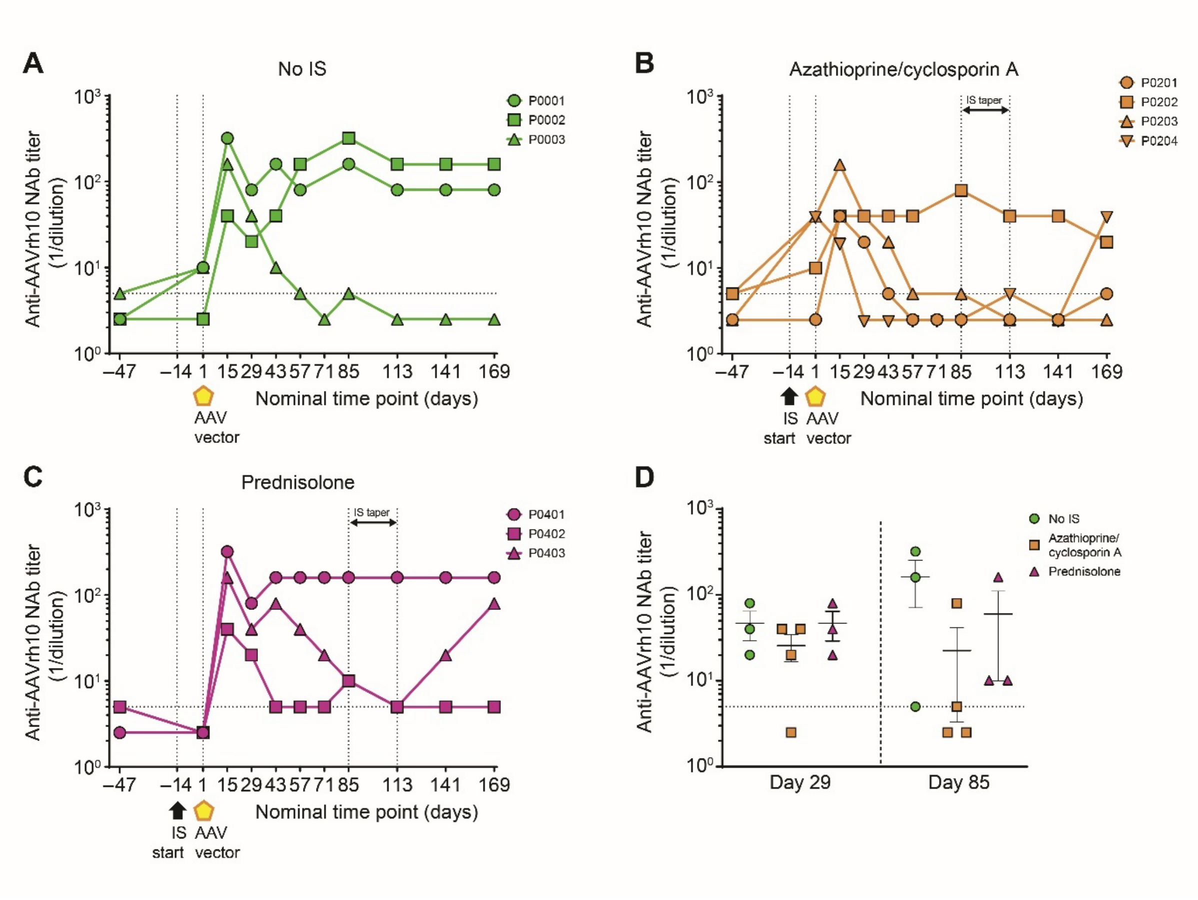

Supplementary Fig. S5. No apparent effect of IS regimens in study 1 on anti-AAVrh10 NAb titers after AAV administration. Serum anti-AAVrh10 NAb titers were evaluated in male cynomolgus macaques using a cell-based neutralizing assay. **A–C** anti-AAVrh10 NAb titers are shown for each individual cynomolgus macaque at all time points analyzed. **D** Anti-AAVrh10 NAb titers at days 29 and 85. Data are represented as mean titer per group ± SEM. The NAb titer values are reported as the reciprocal of the highest serum dilution at which AAV transduction is reduced 50% compared with the negative control. Horizontal dashed lines in **A–D** at anti-AAVrh10 NAb titer (1/dilution) = 5 indicate assay limit of detection; samples below limit of detection are plotted as half the assay limit. Vertical dashed lines in **A–C** at days –14 and 1 indicate start of IS regimen and AAV administration, respectively. Vertical dashed lines in **A–C** at days 85 and 113 indicate IS taper. Statistical analysis in **D** was performed using ordinary one-way ANOVA with Tukey's multiple comparisons test for day 29 and Kruskal-Wallis with Dunn's multiple comparisons test for day 85; all values calculated were $p \geq 0.05$. *AAV* adeno-associated virus, *IS* immunosuppression, *NAb* neutralizing antibody.

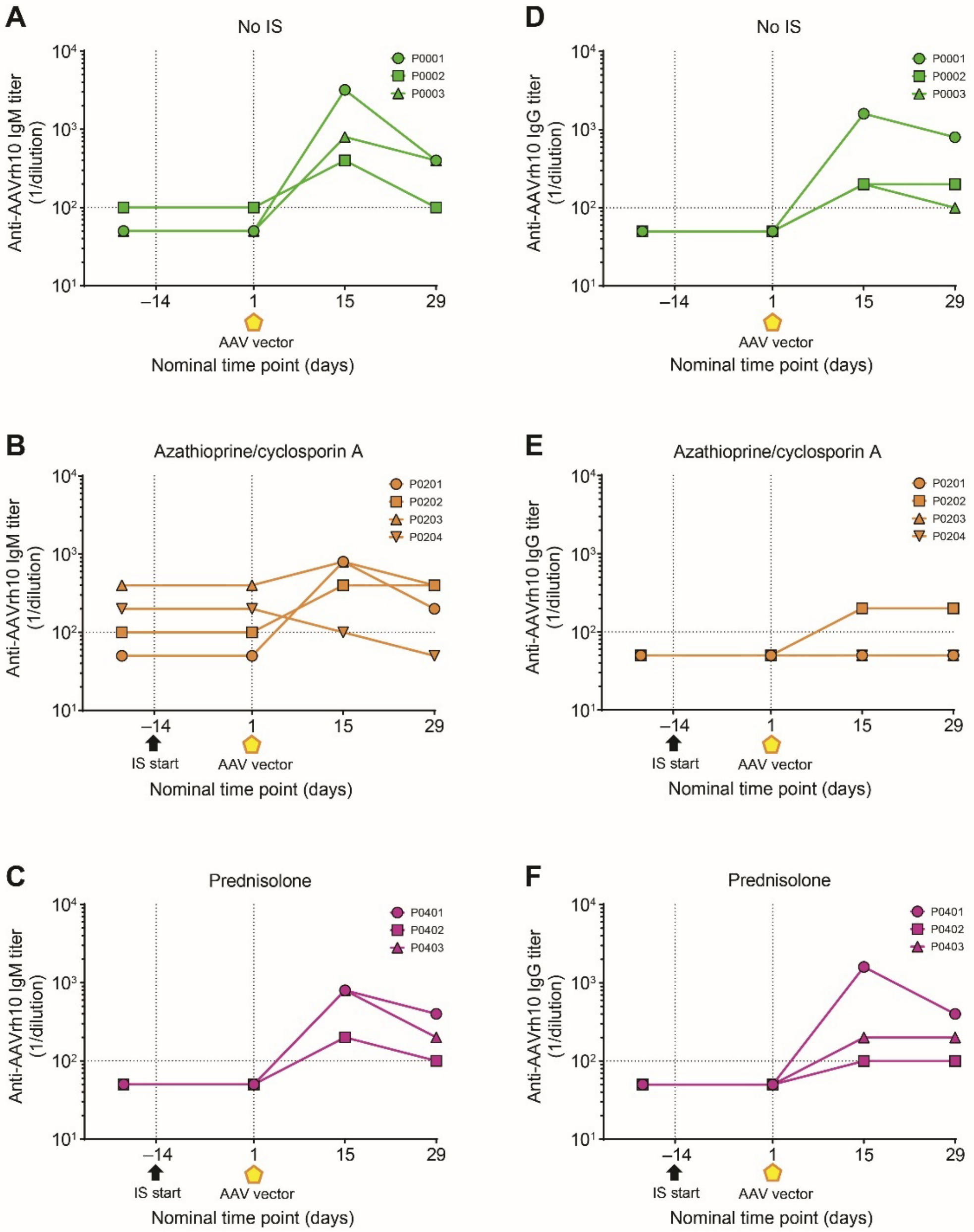

Supplementary Fig. S6. Azathioprine/cyclosporin A reduced anti-AAVrh10 IgG development in study 1. **A–C** Serum anti-AAVrh10 IgM and **D–F** anti-AAVrh10 IgG titers were evaluated in individual male cynomolgus macaques by ELISA at the indicated time points. Horizontal dashed lines at anti-AAVrh10 IgM/IgG titer (1/dilution) = 100 indicates assay limit of detection; samples below limit of detection are plotted as half the assay limit. Vertical dashed lines at days –14 and 1 indicate start of IS regimen and AAV administration, respectively. *AAV* adeno-associated virus, *IS* immunosuppression.

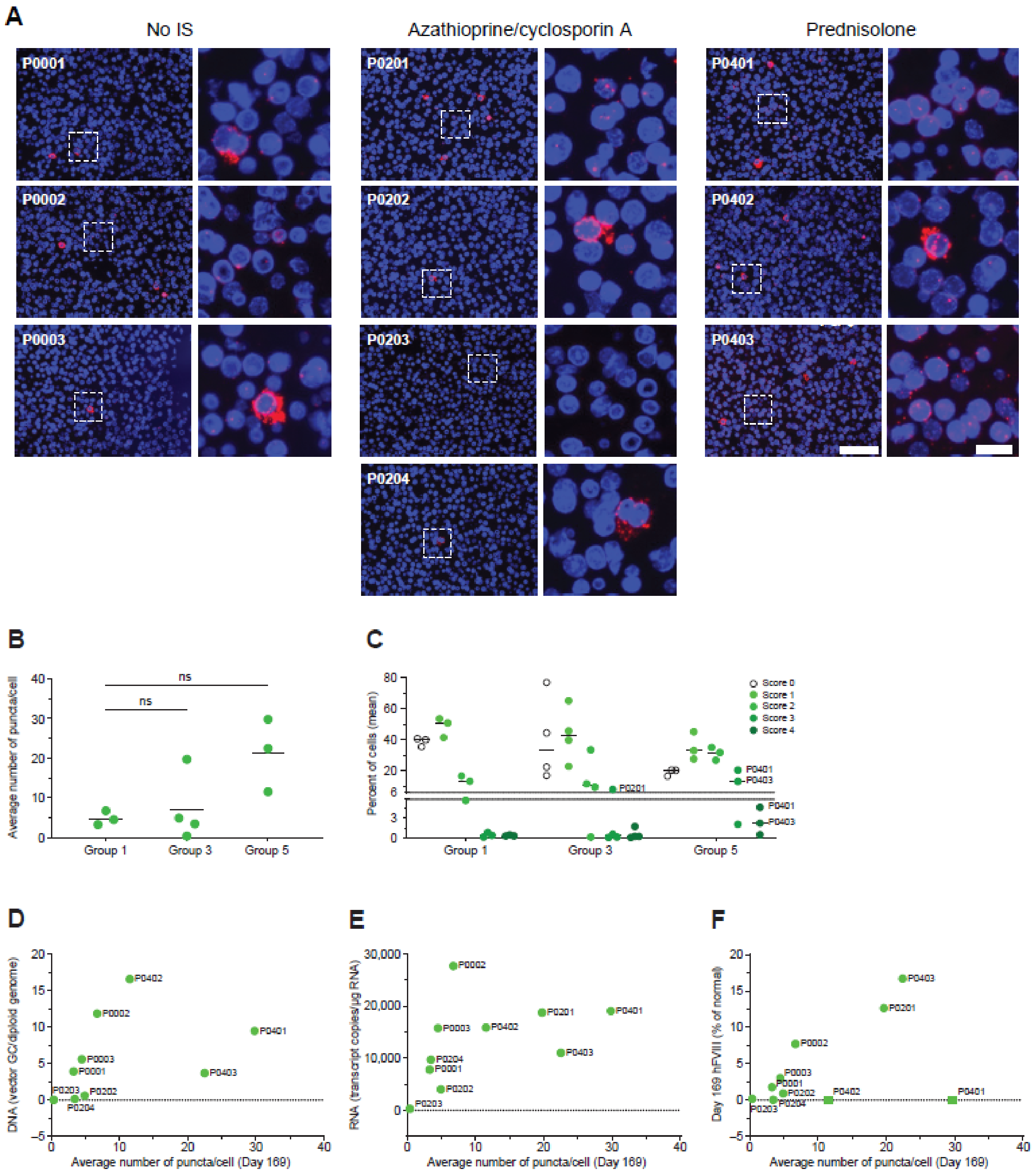

Supplementary Fig. S7. hFVIII expression in the liver by in situ hybridization (ISH). Transgene expression in DAPI+ cells was analyzed in liver sections of individual cynomolgus macaques by performing ISH on day 169. **A** Representative ISH images (20X) are shown for each animal from groups 1 (no IS), 3 (azathioprine/cyclosporin A) and 5 (prednisolone). Scale bar in main figure indicates 100 µm. Scale bar in inset indicates 20 µm. Inset shown to illustrate ISH signal at a higher magnification to more easily visualize cells with low numbers of puncta. **B–C** hFVIII expression was analyzed using image analysis software (HALO; Indica Labs). **B** The average number of puncta from hFVIII hybridization per DAPI+ cell from two images is shown for each individual animal with the group mean shown. **C** Percent of DAPI+ cells in the liver with positive hFVIII hybridization signal by level for individual groups (Score 0 = no hFVIII hybridization signal, Score 1 = 1-9 puncta/cell, Score 2 = 10-49 puncta/cell, Score 3 = 50-99 puncta/cell and Score 4 = ≥100 puncta/cell). Animals P0401, P0403 and P0201 are indicated due to high percentage of score 3 and score 4 cells. **D-F** Comparison of results from day 169 for animals in group 1 (no IS), group 3 (azathioprine/cyclosporin A) and group 3 (prednisolone) for the average hFVIII hybridization signal per animal **D** vs vector GC/diploid genomes

(mean of left and right lobes) **E** vs hFVIII RNA transcript copies (mean of left and right lobes) and **F** vs hFVIII (% of normal) in blood. *GC* genome copies, *IS* immunosuppression.

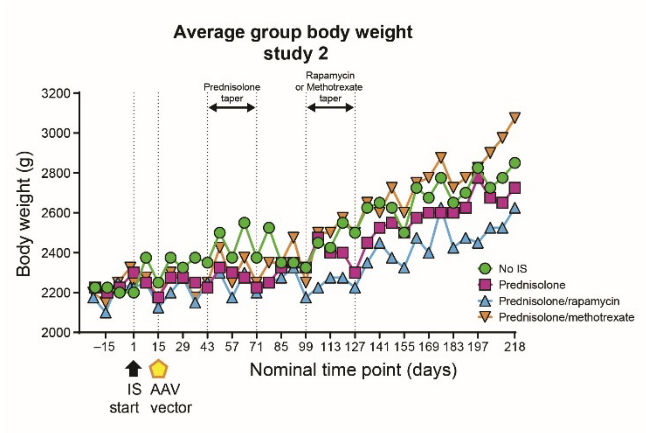

Supplementary Fig. S8. Body weights of study 2 male cynomolgus macaques. Weight in grams was evaluated in individual male cynomolgus macaques at the indicated time points. Data are represented as mean weight for the no IS group and for each IS regimen group. Vertical dashed lines at days 1 and 15 indicate start of IS regimen and AAV administration, respectively. Vertical dashed lines at days 43, 71, 99, and 127 indicate tapering of IS regimens. *AAV* adeno-associated virus vector, *IS* immunosuppression.

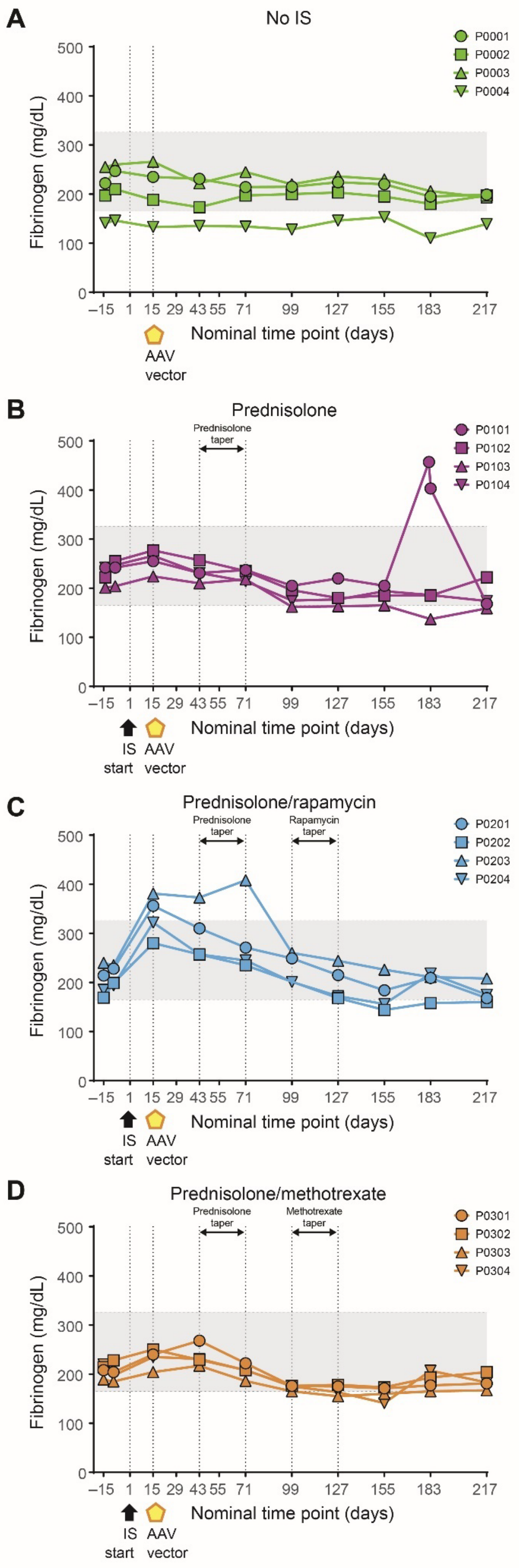
A
No IS
P0001
P0002
P0003
P0004
Fibrinogen (mg/dL)
0
100
200
300
400
500
–15 1 15 29 43 55 71 99 127 155 183 217
Nominal time point (days)
AAV vector
B
Prednisolone
Prednisolone taper
P0101
P0102
P0103
P0104
IS start
AAV vector
C
Prednisolone/rapamycin
Prednisolone taper
Rapamycin taper
P0201
P0202
P0203
P0204
D
Prednisolone/methotrexate
Prednisolone taper
Methotrexate taper
P0301
P0302
P0303
P0304

Supplementary Fig. S9. Plasma fibrinogen in study 2. A–D Plasma fibrinogen was evaluated in individual male cynomolgus macaques using the HemosIL® Fibrinogen-C XL assay at the indicated time points. Horizontal dashed lines and gray area indicates reference range. On days 181–184, animal P0101 experienced transient health issues, including increased fibrinogen levels, that were later attributed to behavioral incompatibility with their cage-mate. Health parameters reverted to normal levels upon repairing animals with new cage-mates on day 184. Vertical dashed lines at days 1 and 15 indicate start of IS regimen and AAV administration, respectively. Vertical dashed lines at days 43, 71, 99, and 127 indicate tapering of IS regimens. *AAV* adeno-associated virus, *IS* immunosuppression.

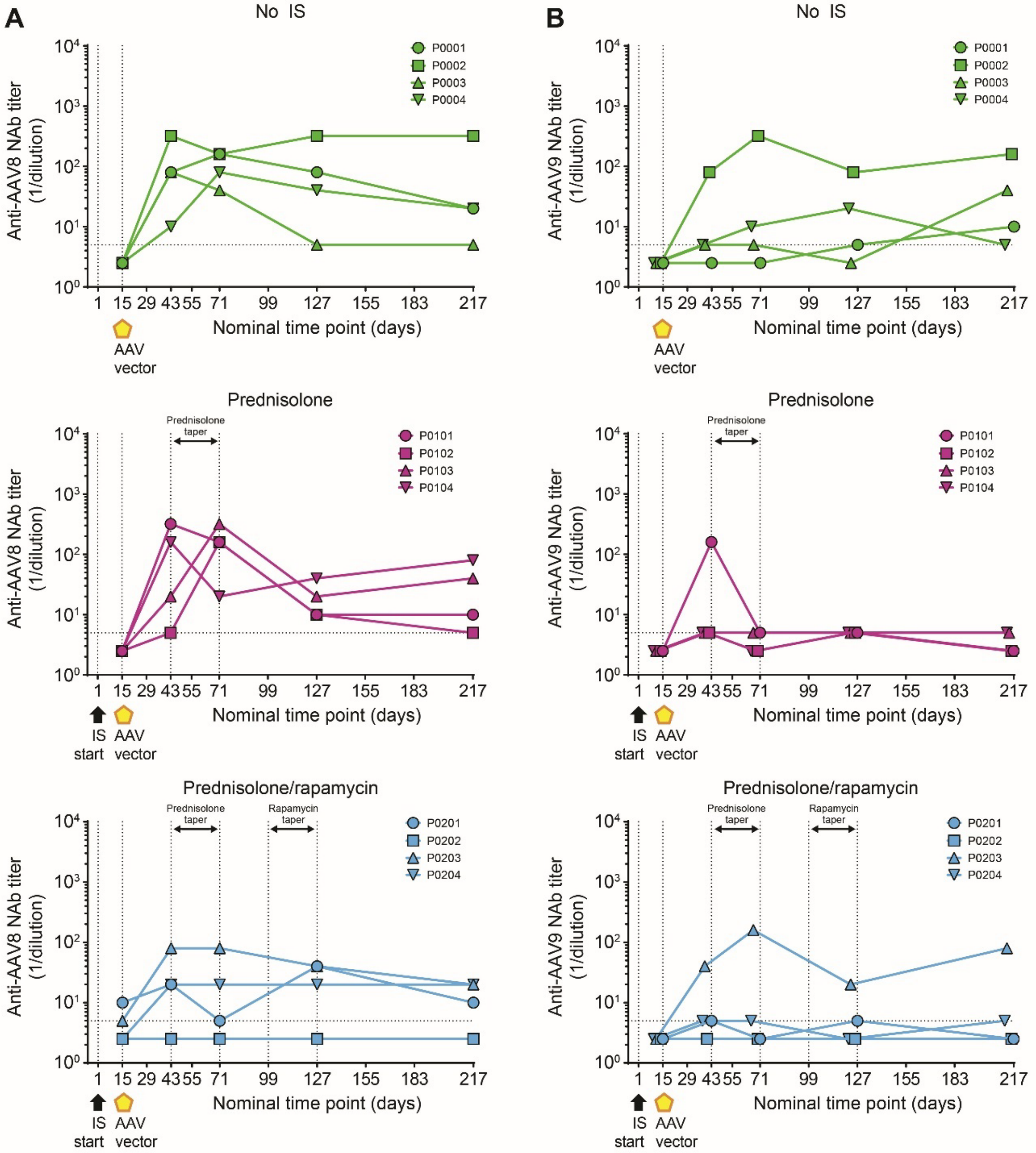

Supplementary Fig. S10. NAb responses to AAV8 and AAV9 in serum samples collected from the no IS, prednisolone, and prednisolone/rapamycin groups of study 2. A Serum anti-AAV8 and **B** anti-AAV9 NAb titers were evaluated in individual male cynomolgus macaques at the indicated time points using a cell-based neutralization assay. The NAb titer values are reported as the reciprocal of the highest serum dilution at which AAV transduction is reduced 50% compared with the negative control. Position of values in **B** has been adjusted slightly along the x-axis for clarity of overlapping datapoints. Horizontal dashed lines at anti-AAV8/AAV9 NAb titer (1/dilution) = 5 indicates assay limit of detection; samples below limit of detection are plotted as half the assay limit. Vertical dashed lines at days 1 and 15 indicate start of IS regimen and AAV administration, respectively. Vertical dashed lines at days 43, 71, 99, and 127 indicate tapering of IS regimens. *AAV* adeno-associated virus, *IS* immunosuppression, *NAb* neutralizing antibody.

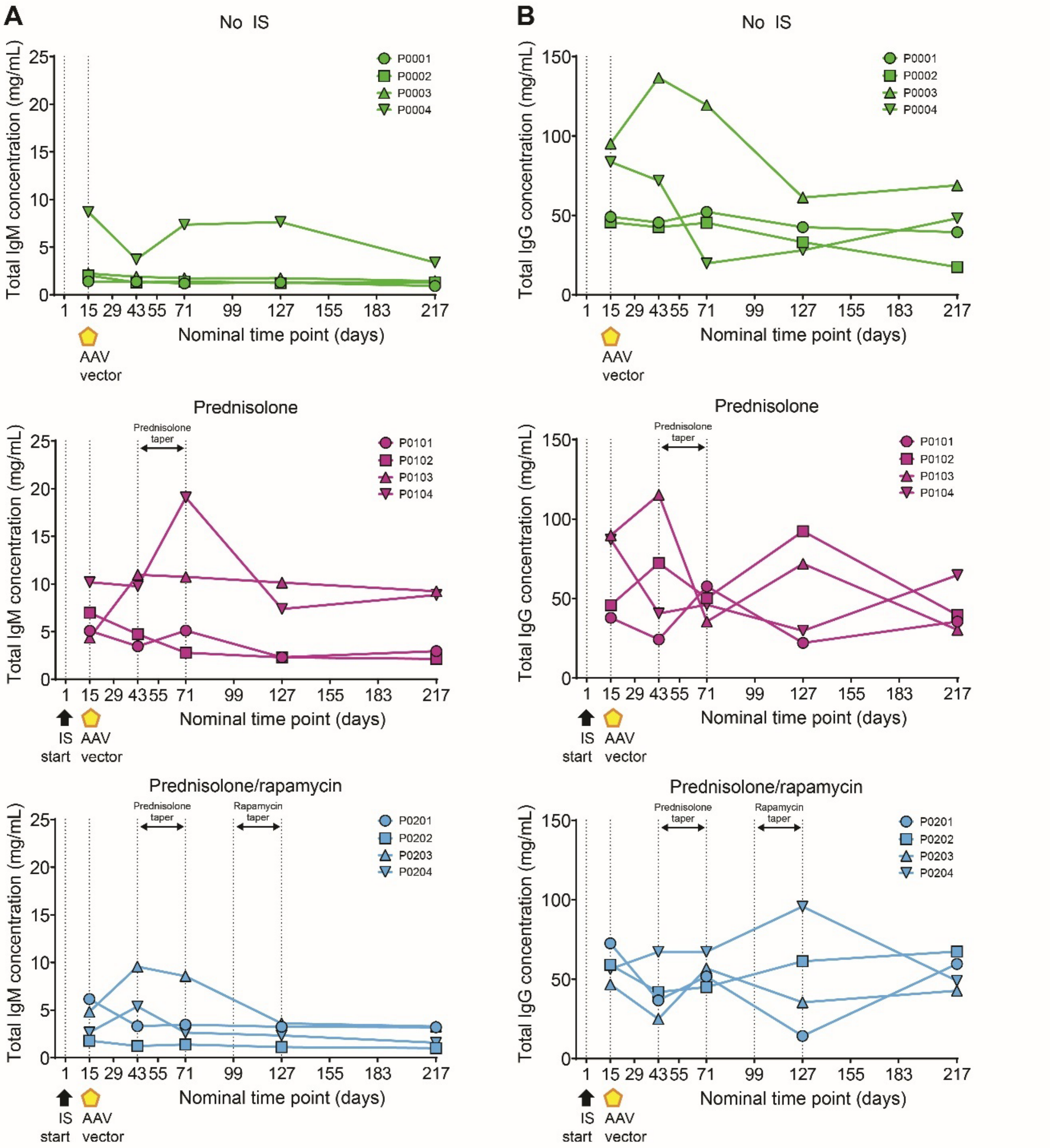

Supplementary Fig. S11. Total IgM and IgG responses in isolated nonhuman primate serum before and after vector administration in study 2. A Concentration of total serum IgM and **B** IgG were evaluated in individual male cynomolgus macaques by ELISA at the indicated time points. Top panel: no IS; middle panel: prednisolone; bottom panel: prednisolone/rapamycin. Vertical dashed lines at days 1 and 15 indicate start of IS regimen and AAV administration, respectively. Vertical dashed lines at days 43, 71, 99, and 127 indicate tapering of IS regimens. *AAV* adeno-associated virus, *IS* immunosuppression.

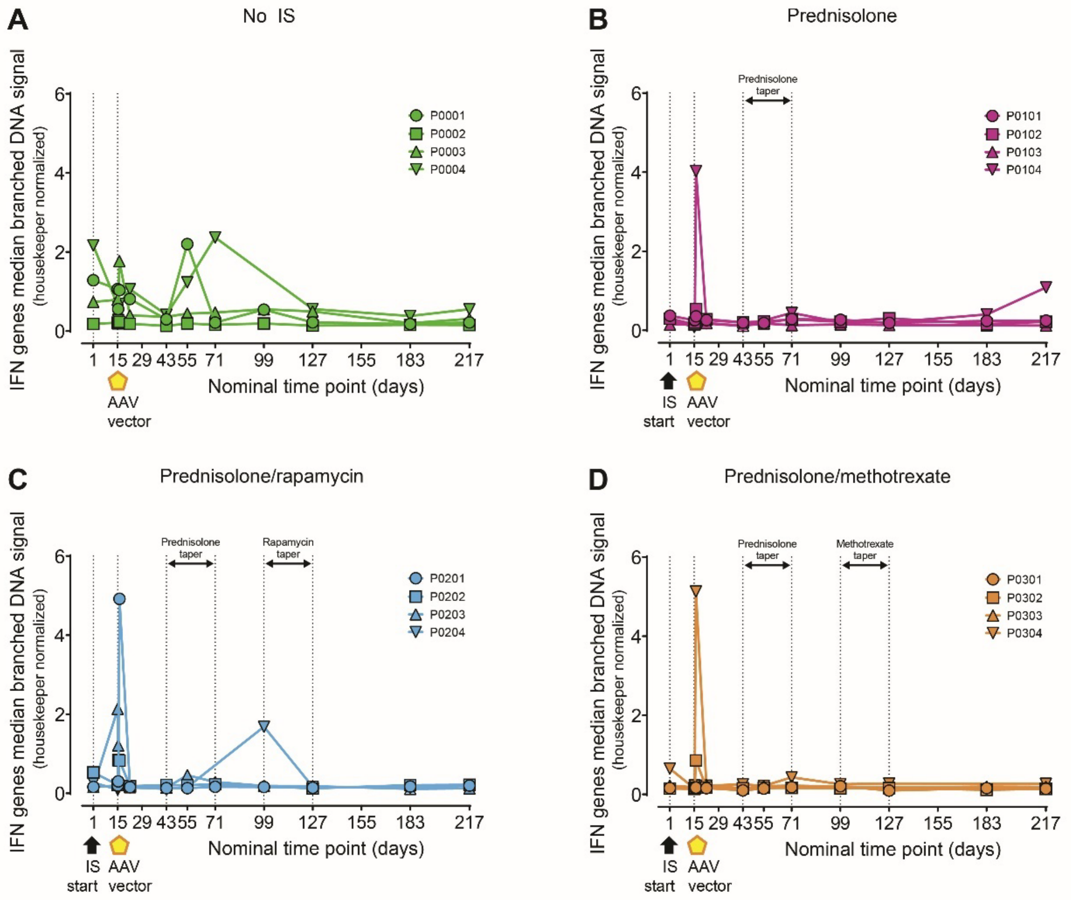
A
No IS
B
Prednisolone
C
Prednisolone/rapamycin
D
Prednisolone/methotrexate
IFN genes median branched DNA signal (housekeeper normalized)
Nominal time point (days)
P0001
P0002
P0003
P0004
P0101
P0102
P0103
P0104
P0201
P0202
P0203
P0204
P0301
P0302
P0303
P0304
Prednisolone taper
Rapamycin taper
Methotrexate taper
IS start
AAV vector

E

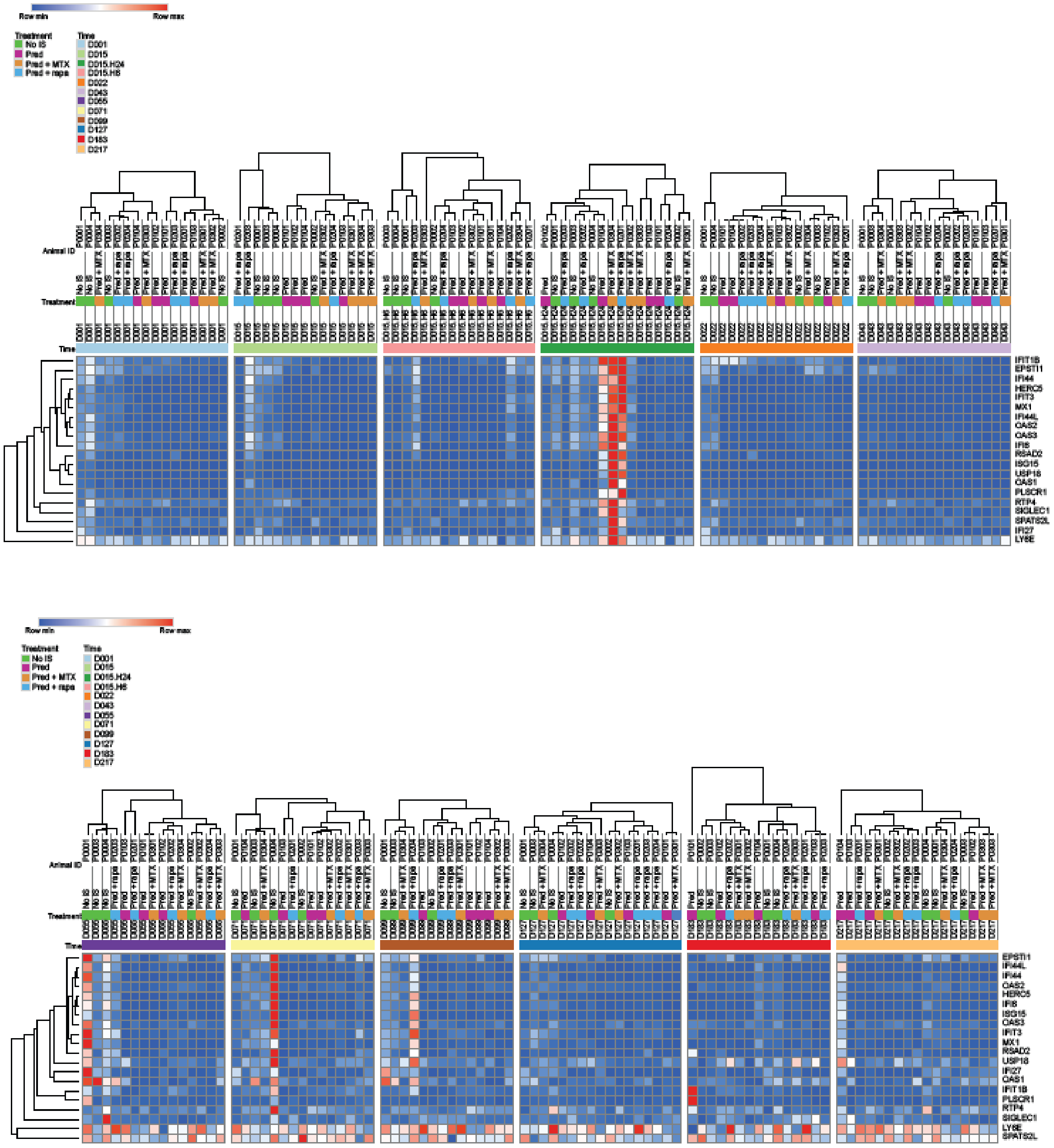

Supplementary Fig. S12. Characterization of IFN gene signature in study 2. The IFN gene signature was evaluated in individual male cynomolgus macaques by determining the expression of 20 genes in whole blood using a branched DNA assay at the indicated time points. Shown are branched DNA signals normalized to expression of housekeeping genes for the **A** no IS, **B** prednisolone, **C** prednisolone/rapamycin group, and **D** prednisolone/methotrexate groups. Vertical dashed lines at days 1 and 15 indicate start of IS regimen and AAV administration, respectively. Vertical dashed lines at days 43, 71, 99, and 127 indicate tapering of IS regimens. **E** IFN gene signature heatmaps showing all 21 genes at days 1, 15 (before, 6 h, and 24 h after vector administration), 22, 43, 55, 71, 99, 127, 183, and 217. See also Supplementary Table S1. *AAV* adeno-associated virus, *D* day, *H* hour, *IFN* interferon, *IS* immunosuppression, *max* maximum, *min* minimum, *MTX* methotrexate, *pred* prednisolone, *rapa* rapamycin.

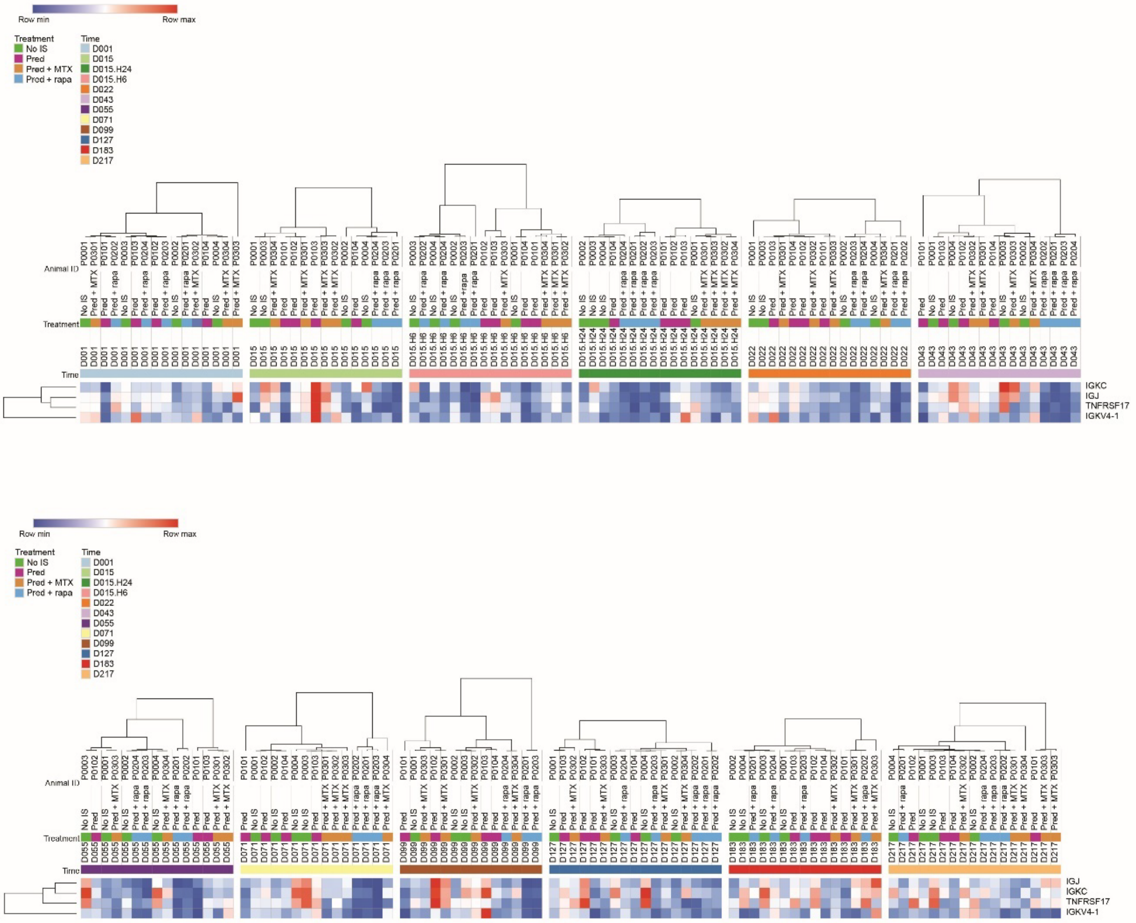

Supplementary Fig. S13. Characterization of PC gene signature in study 2. The PC gene signature was evaluated in individual male cynomolgus macaques by determining the expression of four genes in whole blood using a branched DNA assay at the indicated time points. PC gene signature heatmaps showing all genes at days 1, 15 (before, 6 h, and 24 h after vector administration), 22, 43, 55, 71, 99, 127, 183, and 217. See also Fig. 8. *D* day, *H* hour, *IS* immunosuppression, *max* maximum, *min* minimum, *MTX* methotrexate, *PC* plasma cell, *pred* prednisolone, *rapa* rapamycin.

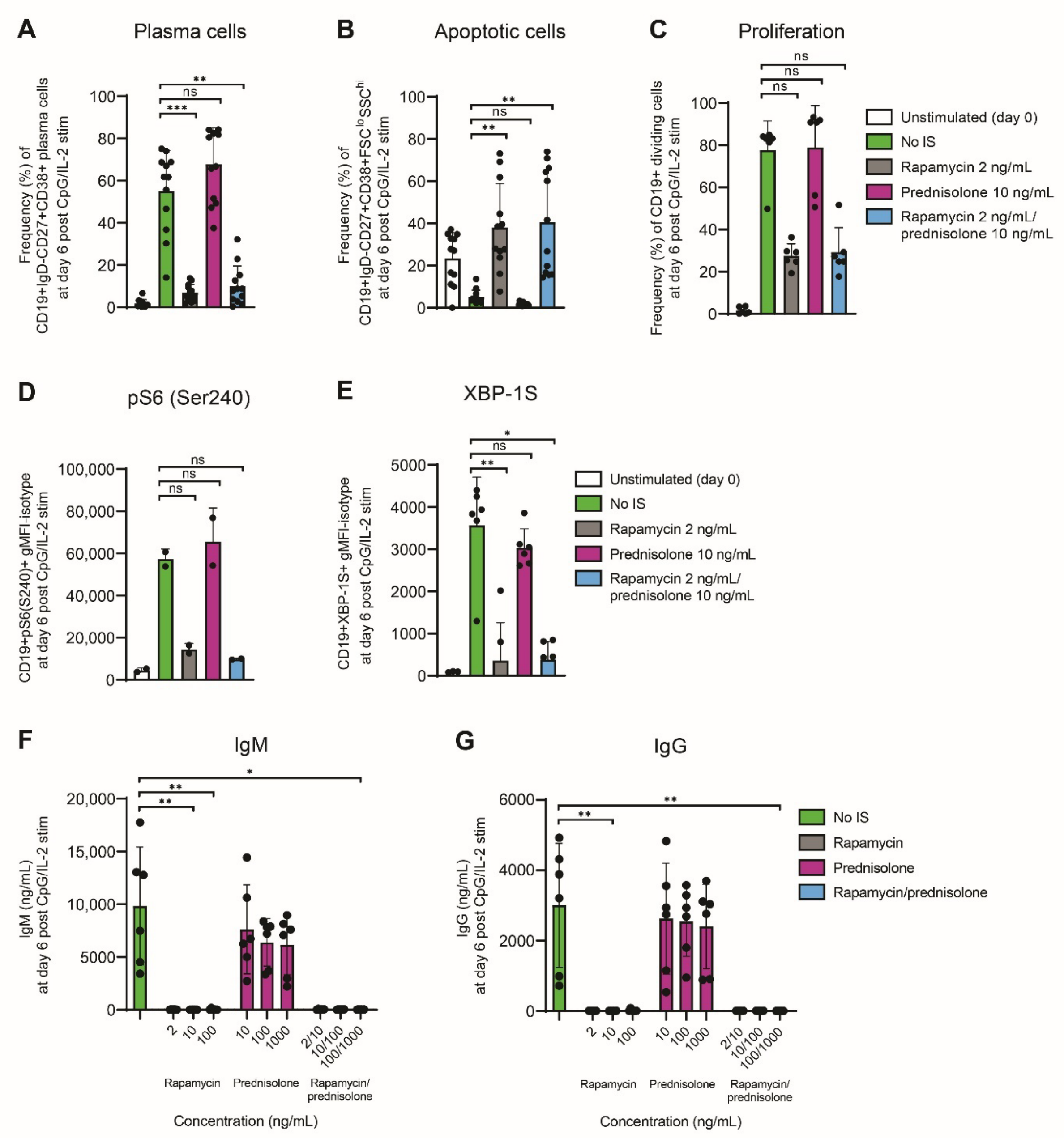

Supplementary Fig. S14. Rapamycin prevents in vitro differentiation of PBMC-derived B cells to PCs. PBMC from three healthy donors were cultured with 1 µg/mL CpG and 100 U/mL IL-2 to induce B-cell differentiation and treated with 2 to 100 ng/mL rapamycin and/or 10 to 1,000 ng/mL prednisolone; unstimulated cells and cells receiving no IS were used as controls. Cells were analyzed after 6 days. **A** Frequency of CD19+IgD-CD27+CD38+ PCs. **B** Frequency of apoptotic ($FSC^{lo}SSC^{hi}$) CD19+IgD-CD27+CD38+ PCs. **C** Frequency of dividing CD19+ cells as measured by CFSE dilution. gMFI of **D** pS6 (Ser240) and **E** XBP-1S in CD19+ cells. ELISA was used to assess **F** IgM and **G** IgG concentration in culture supernatant. Data are represented as mean value per group ± SD of **D** one or **A–C**, **E–G** three donors, with two to four replicates per donor. Statistical analysis in **A–G**) was performed using Kruskal-Wallis test with Dunn's multiple comparisons test; ns $p \geq 0.05$; *$p < 0.05$; **$p < 0.01$; ***$p < 0.001$. *gMFI* geometric mean fluorescence intensity, *IS* immunosuppression, *ns* not significant, *PBMC* peripheral blood mononuclear cell, *PC* plasma cell, *stim* stimulation.

Supplementary Table S1. Proportion of DAPI+ cells with at least one hFVIII puncta in individual animals

Group	Subject #	% DAPI+ cells with at least one hFVIII puncta
1	P0001	59.95
	P0002	59.28
	P0003	64.45
3	P0201	83.03
	P0202	77.55
	P0203	22.94
	P0204	55.50
5	P0401	79.69
	P0402	79.55
	P0403	83.45

Supplementary Table S2. Fold change in IFN gene signature assay signal (IFN/PBS)

Gene	M1 IFN/PBS	Gene	M2 IFN/PBS
ISG15	62.29	*ISG15*	71.07
HERC5	46.02	*IFI44L*	63.41
IFI44L	43.43	*RSAD2*	30.02
MX1	35.50	*HERC5*	27.87
IFIT3	31.78	*IFI44*	27.76
IFI44	30.60	*MX1*	26.31
USP18	26.00	*USP18*	22.65
OAS2	14.22	*IFIT3*	19.03
RSAD2	14.04	*OAS2*	14.29
IFI6	12.46	*SIGLEC1*	11.29
IFI27	10.12	*IFI27*	10.84
PLSCR1	7.35	*OAS1*	10.70
IFIT1B	7.07	*IFI6*	8.89
OAS1	7.07	*EPSTI1*	7.03
OAS3	6.53	*PLSCR1*	5.93
EPSTI1	4.93	*OAS3*	5.89
SIGLEC1	3.89	*IFIT1B*	4.06
SPATS2L	2.26	*SPATS2L*	2.28
LY6E	2.04	*LY6E*	2.05
RTP4	1.92	*RTP4*	2.04

IFN interferon.
